# Neanderthal introgression in *SCN9A* impacts mechanical pain sensitivity

**DOI:** 10.1038/s42003-023-05286-z

**Published:** 2023-10-10

**Authors:** Pierre Faux, Li Ding, Luis Miguel Ramirez-Aristeguieta, J. Camilo Chacón-Duque, Maddalena Comini, Javier Mendoza-Revilla, Macarena Fuentes-Guajardo, Claudia Jaramillo, William Arias, Malena Hurtado, Valeria Villegas, Vanessa Granja, Rodrigo Barquera, Paola Everardo-Martínez, Mirsha Quinto-Sánchez, Jorge Gómez-Valdés, Hugo Villamil-Ramírez, Caio C. Silva de Cerqueira, Tábita Hünemeier, Virginia Ramallo, Rolando Gonzalez-José, Lavinia Schüler-Faccini, Maria-Cátira Bortolini, Victor Acuña-Alonzo, Samuel Canizales-Quinteros, Giovanni Poletti, Carla Gallo, Francisco Rothhammer, Winston Rojas, Annina B. Schmid, Kaustubh Adhikari, David L. Bennett, Andrés Ruiz-Linares

**Affiliations:** 1 Ministry of Education Key Laboratory of Contemporary Anthropology and Collaborative Innovation Center of Genetics and Development, School of Life Sciences and Human Phenome Institute, Fudan University, Yangpu District, 200438 Shanghai, China; 2 UMR ADES, Aix-Marseille Université, CNRS, EFS, 13005 Marseille, France; 3 UMR GenPhySE, INRAE, INP, ENVT, Université de Toulouse, 31326 Castanet-Tolosan, France; 4 QST Lab, Faculty of Odontology, Universidad de Antioquia, 050010 Medellin, Colombia; 5 Centre for Palaeogenetics, Svante Arrhenius väg 20C, SE-10691 Stockholm, Sweden; 6 Department of Archaeology and Classical Studies, Stockholm University, SE-1069 Stockholm, Sweden; 7 Department of Genetics, Evolution and Environment, University College London, London, WC1E 6BT UK; 8 Nuffield Department of Clinical Neurosciences, Oxford University, Oxford, OX3 9DU UK; 9 Laboratorios de Investigación y Desarrollo, Facultad de Ciencias y Filosofía, Universidad Peruana Cayetano Heredia, 31 Lima, Perú; 10 Unit of Human Evolutionary Genetics, Institut Pasteur, 75015 Paris, France; 11 Departamento de Tecnología Médica, Facultad de Ciencias de la Salud, Universidad de Tarapacá, 1000000 Arica, Chile; 12 GENMOL (Genética Molecular), Universidad de Antioquia, 5001000 Medellín, Colombia; 13 Molecular Genetics Laboratory, National School of Anthropology and History, Mexico City, 14050, 6600 Mexico, Mexico; 14 Department of Archaeogenetics, Max Planck Institute for the Science of Human History (MPI-SHH), 07745 Jena, Germany; 15 Forensic Science, Faculty of Medicine, UNAM (Universidad Nacional Autónoma de México), 06320 Mexico City, Mexico; 16 Unidad de Genomica de Poblaciones Aplicada a la Salud, Facultad de Química, UNAM-Instituto Nacional de Medicina Genómica, 4510 Mexico City, Mexico; 17 Scientific Police of São Paulo State, 19900-109 Ourinhos-SP, Brazil; 18 Departamento de Genética e Biologia Evolutiva, Instituto de Biociências, Universidade de São Paulo, 05508-090 São Paulo, SP Brazil; 19 Departamento de Genética, Universidade Federal do Rio Grande do Sul, 90040-060 Porto Alegre, Brasil; 20 Instituto Patagónico de Ciencias Sociales y Humanas, Centro Nacional Patagónico, CONICET, U9129ACD Puerto Madryn, Argentina; 21 Instituto de Alta Investigación, Universidad de Tarapacá, Arica, 1000000 Arica, Chile; 22 School of Mathematics and Statistics, Faculty of Science, Technology, Engineering and Mathematics, The Open University, Milton Keynes, MK7 6AA UK; 23 Department of Cell and Developmental Biology, University College London, London, WC1E 6BT UK

**Keywords:** Population genetics, Ion channels in the nervous system, Genetic association study

## Abstract

The Nav1.7 voltage-gated sodium channel plays a key role in nociception. Three functional variants in the *SCN9A* gene (encoding M932L, V991L, and D1908G in Nav1.7), have recently been identified as stemming from Neanderthal introgression and to associate with pain symptomatology in UK BioBank data. In 1000 genomes data, these variants are absent in Europeans but common in Latin Americans. Analysing high-density genotype data from 7594 Latin Americans, we characterized Neanderthal introgression in *SCN9A*. We find that tracts of introgression occur on a Native American genomic background, have an average length of ~123 kb and overlap the M932L, V991L, and D1908G coding positions. Furthermore, we measured experimentally six pain thresholds in 1623 healthy Colombians. We found that Neanderthal ancestry in *SCN9A* is significantly associated with a lower mechanical pain threshold after sensitization with mustard oil and evidence of additivity of effects across Nav1.7 variants. Our findings support the reported association of Neanderthal Nav1.7 variants with clinical pain, define a specific sensory modality affected by archaic introgression in *SCN9A* and are consistent with independent effects of the Neanderthal variants on Nav1.7 function.

## Abstract (web summary)

Neanderthal-derived variants in the *SCN9A* gene (encoding the voltage gated sodium channel, Nav1.7) are associated with enhanced experimental mechanical pain sensitivity in modern humans.

## Introduction

The sodium voltage-gated channel alpha subunit 9 (*SCN9A*) gene encodes Nav1.7, a sodium channel vital for the generation and conduction of the nerve impulse in nociceptors^1^. Nav1.7 is highly expressed in nociceptive neurons and regulates their excitability through amplification of sub-threshold stimuli, contribution to the rising phase of the action potential and neurotransmitter release at central terminals. Family studies have shown that rare mutations in *SCN9A* can cause Mendelian forms of either insensitivity to pain^2^ or extreme pain syndromes^1,3,4^. Furthermore, association studies have suggested an involvement of *SCN9A* variants in increased pain sensitivity^5^ and small-fibre neuropathy^6^. In 2020, Zeberg et al.^7^ reported that three amino acid substitutions in Nav1.7 (M932L, V991L and D1908G, inferred to stem from Neanderthal introgression) impact on Nav1.7 function in vitro. Furthermore, based on UK Biobank (UKBB) data, Zeberg et al.^7^ found that individuals carrying the three Neanderthal variants manifest greater pain, as reported in the UKBB questionnaire. These Neanderthal variants, however, are rare in UKBB (frequency <0.003), complicating further studies of their role on pain perception in Europeans.

Genetic analysis of clinical pain disorders is of potential clinical relevance. However, interpretation is complex because of the confounding effects of disease type and progression. It is therefore difficult to disentangle whether pain symptomatology results from differences in pain sensitivity, underlying pathology, disease progression (and management), or a combination of these. By contrast, experimental pain models in healthy individuals do not suffer from such high disease confounding. Experimental studies also allow the measurement of pain thresholds to standardized stimuli, in a controlled environment^8^. In the past years, we have been performing genetic analyses of non-disease-related variation in volunteers from Brazil, Colombia, Chile, Peru and Mexico (the CANDELA cohort)^9–14^. This cohort is characterized by its mixed European, Native American and African ancestry, and an extensive genetic and phenotypic diversity^15,16^. Recently, we have been performing genetic studies of experimental pain (using Quantitative Sensory Testing^17^ (QST)) in a sample of Colombian volunteers (the QST cohort). This QST protocol includes assessment of a number of pain thresholds at baseline and then assesses how these change following topical application of mustard oil in order to assess pain sensitization^18^. Mustard oil is an agonist of TRPA1 which is a non-selective cation channel expressed by a sub-population of nociceptive afferents and which is activated by a range of environmental irritants^19^. Topical application of mustard oil results in a rapid activation of nociceptors and sensitization to noxious stimuli. We have previously shown that this sensitization is enhanced in individuals carrying a N855S gain of function mutation in *TRPA1*^20^. Demonstrating the informativity of this approach for the genetic analysis of pain perception, we recently reported the identification of *NCX3* as a gene regulating pain “wind-up” (the temporal summation of pain) through modulation of spinal cord excitability^21^.

Although the Neanderthal *SCN9A* variants studied by Zeberg et al.^7^ are rare in Europeans, they are common in Admixed populations from the Americas (up to a frequency of 0.529 in PEL from 1000 Genomes). Here, we examine Neanderthal introgression in the *SCN9A* region in the CANDELA and QST cohorts. We also evaluate the association of the Neanderthal Nav1.7 variants with increased sensitivity to noxious stimuli in the QST cohort. We find that M932L, V991L and D1908G variants are common in the CANDELA/QST cohorts, being included in Neanderthal introgression tracts extending over ~123Kb in the *SCN9A* region. Furthermore, we find that Neanderthal ancestry in *SCN9A* is significantly associated with a lower mechanical pain threshold after sensitization with mustard oil, with evidence of additive effects across Neanderthal variants. Our findings support the proposal that Neanderthal ancestry in *SCN9A* impacts on pain sensitivity, implicate a specific nociceptive modality and are consistent with independent effects of the Neanderthal variants on Nav1.7 function.

## Results

### Study samples and pain traits examined

We examined two, partially overlapping, population samples. For the analysis of Neanderthal introgression in the *SCN9A* gene region we examined 5971 individuals from the CANDELA cohort (recruited in Brazil, Chile, Colombia, Mexico and Peru). For the analysis of genetic association to experimental pain we examined 1623 Colombians (hitherto referred as: the QST cohort). A total of 710 individuals from the QST cohort are included in the CANDELA cohort (these are Colombian CANDELA volunteers that were recontacted for the collection of pain data). The CANDELA individuals have a mean of 46.0% Native American, 49.6% European and 4.4% African ancestry, but with considerable variation between individuals and countries (Supplementary Data 1 and Supplementary Fig. 1). The QST cohort has a median age of 22 (range 18–45), of which 56.1% are women (Supplementary Data 2). Mean ancestry estimates for this cohort are: 31.3% Native American, 59.0% European and 9.7% African (a similar ancestry distribution as in the Colombians of the CANDELA cohort; Supplementary Data 1 and Supplementary Fig. 1).

Using the protocol described in Schmid et al.^18^, we measured thresholds to heat pain, mechanical pain and pressure pain. We also measured Wind-Up Ratio (WUR; a measure of central sensitization). In addition, heat and mechanical pain thresholds were measured after application of mustard oil (which increases pain sensitivity). We observed a low/moderate but significant correlation of sex with four traits, women manifesting greater pain sensitivity, consistent with previous studies^22,23^. Age and depression scores showed low but significant correlations with two traits (Supplementary Data 3).

### Frequency of Neanderthal M932L, V991L and D1908G Nav1.7 variants in Latin Americans

We imputed individual SNP genotypes for rs3750904, rs4369876 and rs12478318 (encoding respectively variants D1908G, V991L and M932L in Nav1.7) in the combined CANDELA plus QST cohorts (a total of 7594 individuals). Imputation quality scores were consistently high (INFO score >98%) for all three variants. For simplicity, in what follows we refer to these variants using the amino acid terminology, the derived alleles being characteristic of Neanderthals^7^.

In the CANDELA cohort, we observed a frequency for D1908G, V991L and M932L of 0.308, 0.134 and 0.135, respectively (Table 1). Only 6/5971 individuals (0.1%) did not show the co-occurrence of Neanderthal alleles at V991L and M932L, a rate consistent with the imputation concordance rate (the expected proportion of genotypes correctly imputed—estimated to 99.2% for these variants). Nearly identical allele frequencies for the V991L and M932L variants have been reported for all the 1000 Genomes population samples^7^ (Supplementary Data 4). The frequency of the three variants in the Colombians included in the CANDELA and QST cohorts are very similar (respectively, 0.192 v. 0.197 for D1908G and 0.060 v. 0.065 for both V991L and M932L; Table 1).

**Table 1:** Frequency of the D1908G, V991L and M932L Nav1.7 Neanderthal variants in the samples studied here.

Cohort	Country sample	*N*	Mean Native American ancestry	D1908G	V991L	M932L
QST	Colombia	1623	0.311	0.197	0.065	0.065
CANDELA	Brazil	666	0.090	0.049	0.020	0.020
	Colombia	1067	0.293	0.192	0.060	0.060
	Mexico	1216	0.579	0.343	0.225	0.228
	Peru	1257	0.661	0.423	0.185	0.186
	Chile	1765	0.478	0.370	0.124	0.124
	Combined	5971	0.462	0.308	0.134	0.135

**Fig. 1:**
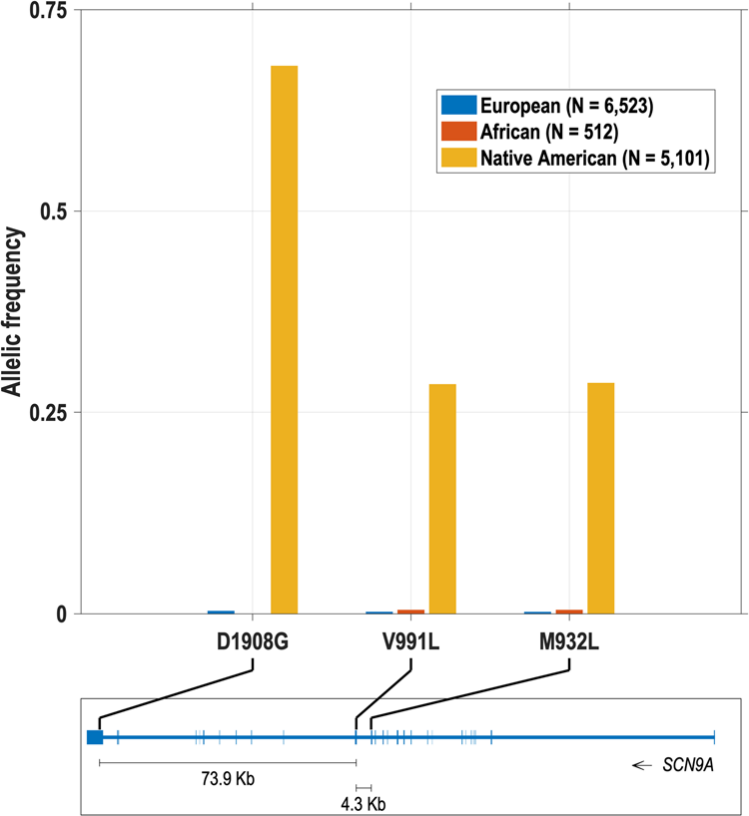
Frequency of the D1908G, V991L and M932L Neanderthal variants in *SCN9A* chromosome segments of Native American, European and African ancestry. These frequencies were estimated on a total of 12,136 chromosomes (from both the QST and CANDELA cohorts). Additional information on these segments is provided in Table 2. The bottom panel shows the location of the D1908G, V991L and M932L variants in *SCN9A* (exons are indicated as boxes/vertical lines) using GRCh37 (hg19) genomic coordinates of human chromosome 2 (physical coordinates 2:167,051,695–167,232,511).

In the CANDELA cohort the frequency of Neanderthal alleles increases with the Native American ancestry proportion of the sample examined (Table 1). For D1908G, highest frequency was observed in Peru (0.423) and lowest in Brazil (0.049), which are the country samples with highest and lowest Native American ancestry (0.66 and 0.09 respectively). A similar trend is observed for V991L and M932L (Table 1). In order to directly relate Neanderthal introgression to continental ancestry we aligned the phased genotypes encoding D1908G, V991L and M932L to local chromosomal ancestry in the *SCN9A* region (inferred using RFMix v1^24^). The three Neanderthal alleles are essentially absent in European and African chromosome segments, while in Native American chromosome segments the frequency of D1908G is 0.680 and that of V991L and M932L is 0.290 (Fig. 1).

There is very strong linkage disequilibrium (LD) between D1908G, V991L and M932L. In the CANDELA cohort we estimate an *r*^2^ of 0.996 and a *D*’ of 1 between V991L and M932L, indicating no recombination between these two variants and nearly identical allele frequencies (as indicated above and Table 1). Between D1908G and the other two variants we observe an *r*^2^ of 0.22 and a *D*’ of 0.80, indicating low recombination and a considerable difference in allele frequencies (as seen in Table 1). Similar values of LD are observed in the QST cohort (Supplementary Data 5). The strong LD between these variants reflects their proximity in *SCN9A*: 73.9 Kb separating D1908G and V991L, and 4.3 Kb separating V991L and M932L (Fig. 1).

We used phased data to estimate the frequency of 3-variant haplotypes (ordered: D1908G-V991L-M932L). Four haplotypes represent >99.9% of chromosomes in the CANDELA sample (similar observations were made in the QST cohort; Supplementary Data 6): the fully ancestral haplotype (D-V-M) is the most common, with a frequency of 0.671, while the fully Neanderthal haplotype (G-L-L) has a frequency of 0.114. In addition, we infer a haplotype carrying a Neanderthal allele only at D1908G (G-V-M) with a frequency of 0.192 and another haplotype carrying Neanderthal alleles only at V991L and M932L (D-L-L), with a frequency of 2.1%. These 3-variant haplotype frequencies mirror the LD and allele frequency estimates mentioned above. The four haplotypes we observe in the CANDELA and QST cohorts are also the only haplotypes seen in the whole genome sequence data of the 1000 Genome Project Phase III (1000GP3^25^; Supplementary Fig. 2 and Supplementary Data 6).

### Neanderthal introgression in the *SCN9A* region

**Fig. 2:**
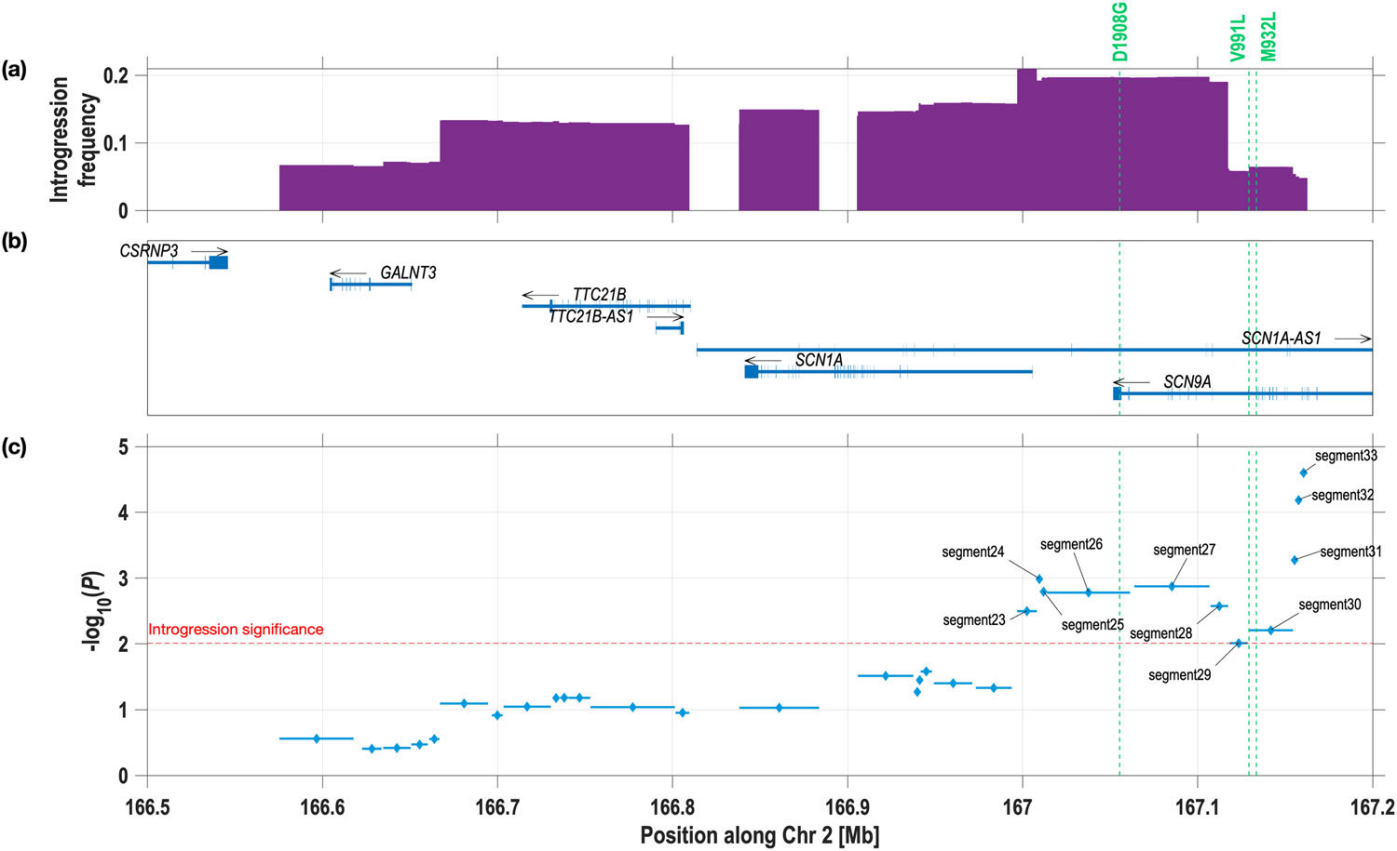
Neanderthal introgression around *SCN9A* and association with mechanical pain threshold after sensitization by mustard oil (POST_MPT) in the QST cohort. Only 700 Kb of the 4.18 Mb studied around *SCN9A* (Table 2) are shown as this region shows the maximum levels of introgression. This region covers the physical coordinates 2:166,500,000-167,200,000 of the GRCh37 (hg19) reference genome. The location of the D1908G, V991L and M932L Neanderthal variants is indicated with vertical dashed lines. The top panel (**a**) shows (in violet) the frequency of Neanderthal introgression tracts (piling-up tracts across individuals). A similar profile is seen in the CANDELA cohort (Supplementary Fig. 3). The middle panel (**b**) shows the location of genes in the region with boxes/vertical lines indicating exons (physical coordinates obtained from GENCODE version 34). The bottom panel (**c**) shows association *P* values of Neanderthal introgression segments with POST_MPT (on *n* = 1552 independent samples). The threshold for significance is indicated by a dashed red line (*P* = 9.75 × 10^−3^, corrected for multiple testing). Significantly associated segments are labelled (further details on these segments are provided in Supplementary Data 8).

We scanned for Neanderthal introgression in the genome of 7594 individuals across a 4 Mb region centred around *SCN9A* (a total of 15,188 chromosomes, comprising the CANDELA and QST cohorts). Retaining only tracts with length ≥23.5 Kb (see Supplementary Note 1 for details), we inferred 12,220 Neanderthal tracts, with an average length of ~123 Kb, corresponding to an average proportion of Neanderthal ancestry in the region of 1.83% in the QST cohort and 2.51% in the CANDELA cohort (Table 2). We examined separately the 12,136 chromosomes that uniformly have only one continental local ancestry across the *SCN9A* region analysed (either African, European or Native American). We found that introgression was detected almost exclusively in chromosomes of Native ancestry (Fig. 1 and Table 2). On average, 5.62% of the DNA for this region stems from Neanderthal in Native American chromosomes (i.e. higher than the 1.37% genome average previously reported in Native Americans^26^). Consistent with a Native American origin for the Neanderthal introgression tracts seen in Latin Americans, the proportion of Neanderthal DNA in the region examined mirrors the average Native American ancestry estimated for each country sample (as seen for the frequencies of D1908G, V991L and M932L, Tables 1, 2). The rare observation of Neanderthal tracts in European and African chromosomes (0.03% and 0.01% frequency, respectively) probably represent phasing and local ancestry inference inaccuracies. Figure 2a displays the 700 Kb genome segment with highest Neanderthal introgression across the 4 Mb region studied in the QST cohort. This segment overlaps the 3’end of *SCN9A* and includes the D1908G, V991L and M932L variants. Introgression peaks around D1908G, with ~20% of chromosomes of the QST cohort carrying a ~100 Kb Neanderthal tract including this variant (consistent with the 0.197 frequency of D1908G in this cohort, Table 1). Similar observations are seen in the CANDELA cohort (Supplementary Fig. 3).

**Table 2:** Features of Neanderthal introgression tracts in the *SCN9A* region^a^.

Study sample	# Chromosomes	# Neanderthal tracts	Mean length (Kb) of introgressed tracts (SE)	Mean % of Neanderthal DNA in region (SE)
QST cohort	3246	2005	124.1 (1.8)	1.83 (0.07)
CANDELA cohort	11,942	10,215	122.6 (0.8)	2.51 (0.04)
Brazil	1332	214	113.4 (5.0)	0.44 (0.05)
Chile	3530	3622	124.7 (1.3)	3.06 (0.07)
Colombia	2134	1293	124.5 (2.2)	1.80 (0.08)
Mexico	2432	2247	117.1 (1.5)	2.59 (0.07)
Peru	2514	2839	124.3 (1.4)	3.36 (0.08)
Chromosome segments of single ancestry^b^				
African	512	4	37.8 (6.8)	0.01 (0.00)
European	6523	104	81.9 (5.1)	0.03 (0.01)
Native American	5101	9666	124.0 (0.8)	5.62 (0.06)

^a^The region examined comprises *SCN9A* ± 2 Mb (i.e. from 165.05 to 169.23 Mb, 4.18 Mb in total, including the 700 Kb region shown in Fig. 2).

^b^Including both the QST and CANDELA cohorts.

### Neanderthal ancestry in the *SCN9A* region and experimental pain traits

We examined the QST cohort to evaluate association between pain traits and Neanderthal ancestry in *SCN9A* using five types of tests: (i) single locus tests (for the Nav1.7 Neanderthal variants), (ii) a joint test of these variants, (iii) haplotype-based tests, (iv) summing the number of Neanderthal alleles across loci and (v) regional introgression analyses. Given the essentially perfect LD between V991L and M932L, for the first four tests, we considered these two variants as a single locus (i.e. V991L-M932L, representing the co-occurrence of Neanderthal alleles at V991L and M932L in 99.9% of the QST chromosomes). For all tests we used a linear regression approach (adjusting for covariates) and considered a study-wide multiple-testing-adjusted significance threshold *P* value of 9.75 × 10^−3^ (see “Methods”). This approach was justified by the nature of the QST phenotypes, which, unlike those tested in Zeberg et al.^7^, are quantitative.

**Fig. 3:**
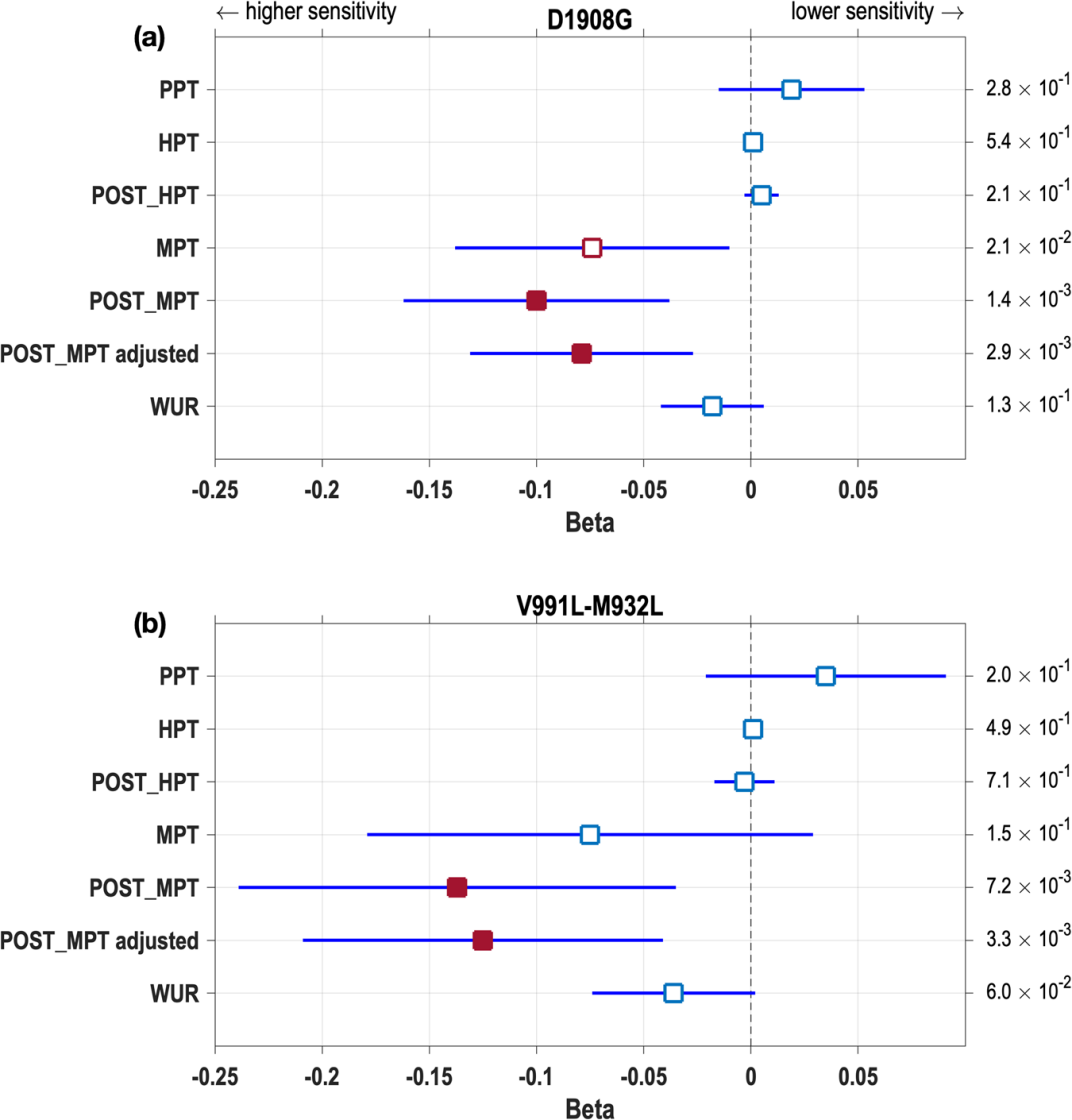
Effect (Beta regression coefficients) of Neanderthal Nav1.7 variants on the pain thresholds evaluated in the QST sample. **a** D1908G, **b** V991L-M932L (considered as a single locus, as indicated in the text). Association *P* values from the regression analysis (on *n* = 1623 independent samples) are reported on the right of each panel. Filled red squares indicate Betas exceeding the multiple-testing-corrected significance threshold (*P* value < 9.75 × 10^−3^). Empty red squares are nominally significant (*P* value < 5 × 10^−2^). Blue lines indicate 95% confidence intervals of Betas. PPT pressure pain threshold, HPT heat pain threshold, MPT mechanical pain threshold, WUR wind-up ratio, POST pain threshold after sensitization with mustard oil, POST_MPT adjusted POST_MPT adjusted for MPT.

We first used the single-locus tests to evaluate association with the six quantitative pain traits examined in the QST cohort. We observed that both D1908G and V991L-M932L are significantly associated with mechanical pain threshold after sensitization with mustard oil (“POST_MPT”, Fig. 3). Association was most significant with D1908G (*P* < 1.4 × 10^−3^), consistent with the higher frequency of this variant. D1908G is also associated with mechanical pain before sensitization (MPT), but only at nominal significance (Fig. 3a; *P* = 2.2 × 10^−2^). We therefore also tested association with POST_MPT adjusted for MPT. This was found to be also significant (Fig. 3a). In line with Zeberg et al.^7^, for all significant associations, the allelic effects are consistently towards increasing pain sensitivity (negative Beta regression coefficients, Fig. 3). The Beta coefficients are of similar magnitude for the two loci tested, although slightly higher for V991L-M932L, relative to D1908G.

**Fig. 4:**
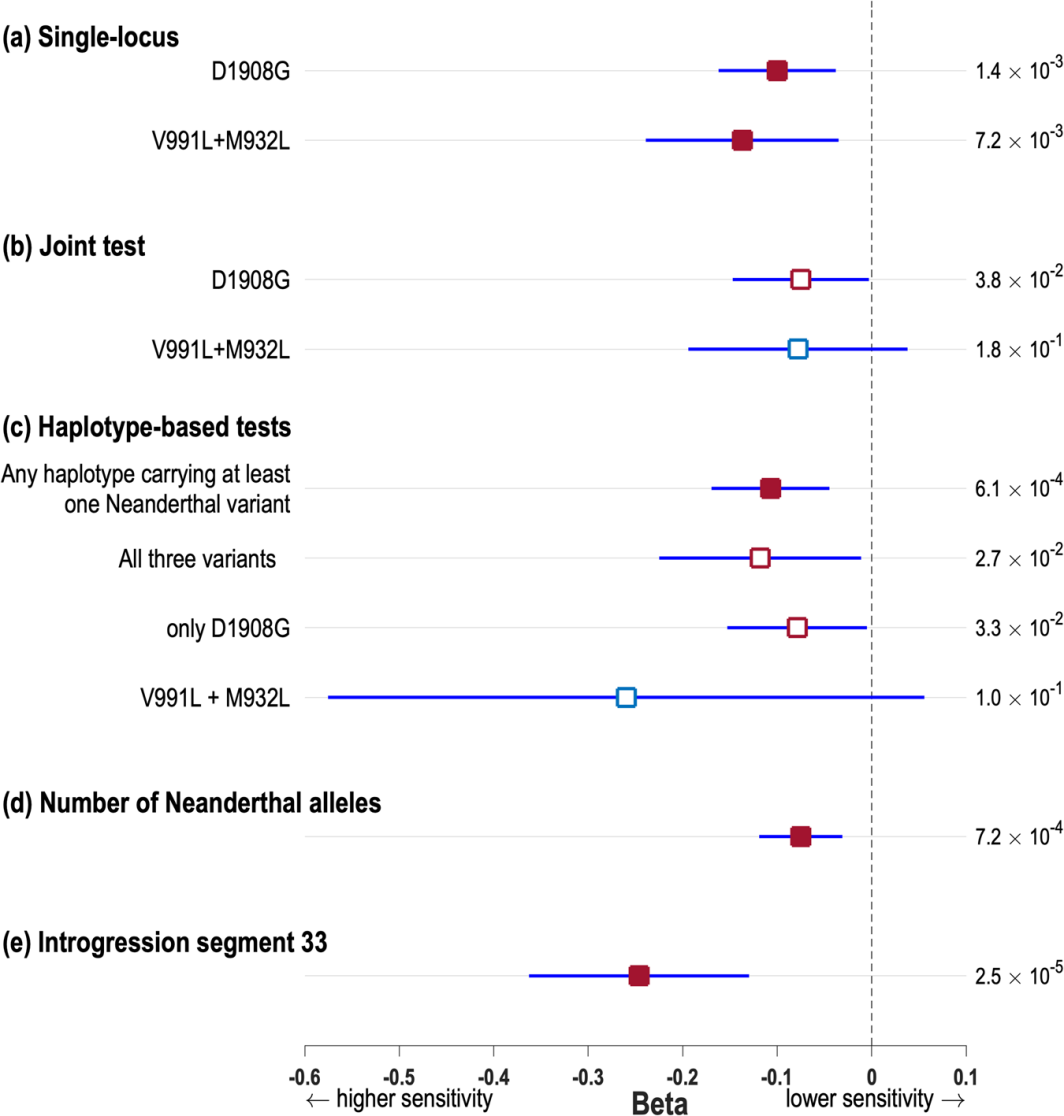
Five types of association tests of Neanderthal ancestry in the *SCN9A* region with mechanical pain threshold after sensitization with mustard oil (POST_MPT). The squares show the Beta regression coefficients for each test of association (estimated on *n* = 1552 independent samples); their *P* values are reported on the right of each panel. Filled red squares indicate Betas exceeding the multiple-testing-corrected significance threshold (*P* value < 9.75 × 10^−3^). Empty red squares are nominally significant (*P* value < 5 × 10^−2^). Blue lines indicate 95% confidence intervals of Betas. **a** Effect of D1908G or V991L-M932L in single-locus tests (Fig. 3). **b** Effects of D1908G and of V991L-M932L in a joint test including these two loci. **c** Effects of the four possible haplotypes carrying Neanderthal variants at either loci (the haplotype not carrying any Neanderthal variant is shown as its complementary: any haplotype carrying at least one Neanderthal variant). **d** Effect of the total number of Neanderthal alleles at D1908G and V991L-M932L (Fig. 5). **e** Effect of Neanderthal introgression segment 33 (from Fig. 2; test results for other introgression segments are shown in Supplementary Data 8).

**Fig. 5:**
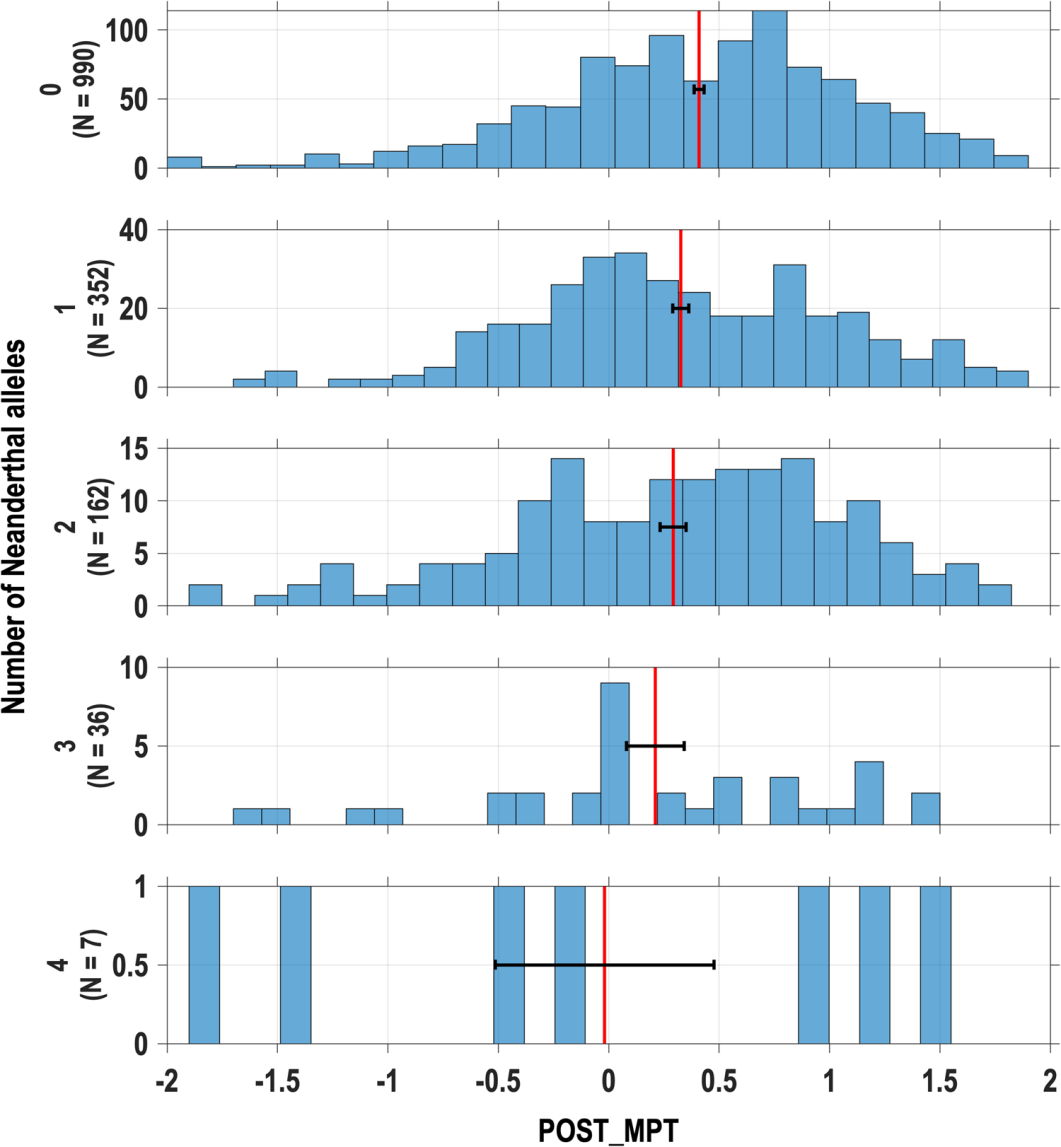
Total number of Neanderthal alleles at D1908G and V991L-M932L, and mechanical pain threshold after sensitization with mustard oil (POST_MPT). Distribution of POST_MPT values for each value of total number of Neanderthal alleles at the two loci (0–4—sample sizes are given between parentheses). Red vertical lines indicate the mean POST_MPT for individuals with 0–4 Neanderthal alleles (horizontal black whiskers indicate standard errors). The trend observed is significant: regression Beta = −0.075, *P* value < 7.2 × 10^−4^ (reported in Fig. 4d).

Since the single-locus tests implicate mainly POST_MPT we continued to examine the effect of Neanderthal introgression in *SCN9A* only for that trait. An issue of special interest is whether the three Neanderthal variants all impact on pain sensitivity. In single-locus tests, the strong LD between D1908G and V991L-M932L complicates an assessment of the possibility that both these loci could impact on POST_MPT (LD makes impossible a distinction between V991L and M932L in these tests). The phased data show that ~90% of the chromosomes carrying Neanderthal alleles at V991L-M932L also carry a Neanderthal allele at D1908G. Conversely, only ~30% of chromosomes carrying a Neanderthal allele at D1908G also carry Neanderthal alleles at V991L-M932L. Consistent with this strong LD, in a joint test (i.e. including in the linear model two genotypic terms, one for each locus) the effect of D1908G drops to nominal significance (*P* value = 3.8 × 10^−2^; Fig. 4b and Supplementary Data 7), and that of V991L-M932L becomes not significant. Since haplotypes reflect the LD between D1908G and V991L-M932L we evaluated association with the four haplotypes found in the QST cohort. As described above, two of these represent the fully ancestral and the fully derived (Neanderthal) haplotypes, while two carry Neanderthal alleles at one locus but not the other. We performed four binary tests, contrasting each individual haplotype against the set including the other three (Fig. 4c and Supplementary Data 7). The contrast between the set including haplotypes carrying one or more Neanderthal alleles to the fully ancestral haplotype showed significant association (actually the most significant association of our study; see Supplementary Data 7), consistent with all haplotypes carrying Neanderthal alleles having an effect on POST_MPT. Although nominally significant, the triple-variant haplotype has a larger effect than the haplotype carrying only D1908G, thereby suggesting an additive effect of both loci. A direct way to evaluate the possibility that both D1908G and V991L-M932L impact on POST_MPT (accounting for the LD between the loci) is to test for an association with the total number of Neanderthal alleles carried by an individual (i.e. summing across loci). This test shows a negative, significant, trend (*β* = −0,075, *P* value = 7.2 × 10^−4^, Figs. 4 and 5 and Supplementary Data 7), consistent with allelic effects being additive across D1908G and V991L-M932L.

Finally, the regional introgression analyses described above allow an exploration of association of Neanderthal ancestry with POST_MPT across the *SCN9A* region, beyond the three known amino acid substitutions. We observe an increase in the significance of association for Neanderthal segments towards the right end of the region of introgression, variant D1908G being included in segment 26 and variants V991L and M932L in segment 30 (Fig. 2 and Supplementary Data 8). Interestingly, strongest association is seen for two short, non-coding segments (Fig. 2). In agreement with the single-variant and haplotype analyses, the effect of introgressed segments is consistently to increase pain sensitivity (negative Betas; Fig. 4 and Supplementary Data 8).

## Discussion

Zeberg et al.^7^ reported that individuals carrying the three Neanderthal Nav1.7 variants (M932L, V991L and D1908G) significantly over-reported a broad clinical pain phenotype (i.e. any of 19 forms of pain recorded in the UKBB questionnaire). However, individuals carrying these three variants are rare in Europe (frequency of 0% in 1000 Genomes and 0.2 % in the UKBB data). The rarity of these variants in Europeans complicates further analysis of their potential impact on pain sensitivity in this population. Furthermore, the UKBB pain data does not provide information into specific sensory modalities that could be impacted by Neanderthal introgression in *SCN9A*. Here we aimed to overcome these limitations of the Zeberg et al.^7^ study by: (i) examining a population with a relatively high frequency of the Neanderthal Nav1.7 variants and (ii) defining sensory phenotypes experimentally, and in individuals with no clinical pain symptomatology.

In agreement with 1000 Genomes data, we observe that M932L, V991L, and D1908G are relatively common in admixed Latin Americans, the frequency of these variants reflecting the extent of Native American admixture in the samples examined (Table 1). Our analysis of *SCN9A* genome segments with only Native American, European or African ancestry is consistent with the view that the Nav1.7 Neanderthal alleles trace their origin to the Native Americans that have contributed to the admixture in the Latin American populations examined. We infer that the average allele frequency of D1908G in these Native Americans was 0.680 and that of V991L and M932L 0.290 (Fig. 1), in line with frequencies observed in contemporary non-admixed Native American populations (0.659 and 0.291, respectively; Supplementary Data 4). These allele frequencies are by far the highest reported anywhere in the world. In 1000 Genomes data, other than in admixed Latin Americans (AMR), maximal frequencies are observed in East Asians, but these never exceed 0.177 for D1908G or 0.078 for and V991L and M932L (Supplementary Data 4). The high frequency of these variants in Native Americans probably relates to strong drift during the initial colonization of the American continent^27,28^.

With regards to nociception, our analyses support the report by Zeberg et al.^7^ of an effect of Neanderthal ancestry in *SCN9A* on pain sensitivity. Furthermore, we find that a particular pain modality impacted by Neanderthal ancestry is mechanical pain sensitivity, especially after mustard oil sensitization (POST_MPT). Given the high expression of Nav1.7 in sensory neurons this is the most likely locus of action of the Neanderthal variants. Mustard oil activates the cation channel TRPA1 (which responds to environmental irritants^29^) expressed on nociceptors so sensitizing these neurons. In addition, we found that the significant association with this post-sensitization phenotype survived correcting for the pre-sensitization phenotype (mechanical pain threshold measured prior to sensitization by mustard oil), therefore indicating that the focal phenotype could be algogenous sensitization rather than mechanical pain itself. Accounting for the baseline mechanical pain actually strengthens the significance for V991L and M932L (adjusted POST_MPT in Fig. 3). Our results suggest that Neanderthal variants further enhance excitability which would be consistent with the role of Nav1.7 in amplifying sub-threshold potentials although effects on action potential propagation or neurotransmitter release in the dorsal horn cannot be excluded. The fact that we saw selective effects on mechanical pain is interesting and distinct Nav1.7 variants have been linked to particular sensory phenotypes, for instance Nav1.7 variants causing inherited erythromelalgia (IEM) are associated with enhanced thermal pain^30^ whilst, those causing paroxysmal extreme pain disorder with enhanced mechanical pain^4^ in the sacral and mandibular regions and painful neuropathy enhanced pressure pain^31^. This may relate to distinct sensory neuron sub-types mediating particular sensory modalities and the impact of environment of the channel (for instance, warmth can enhance the hyperpolarizing shift in activation of IEM causing mutations^32^).

In their report, Zeberg et al.^7^ observed a significant association with pain only for individuals carrying the three Neanderthal alleles (M932L, V991L and D1908G). They reported no association for individuals carrying only one or two Neanderthal alleles (in various combinations). The possibility that these alleles could have independent effects is of considerable importance considering experimental evidence on their functional impact on channel function, individually or in various combinations. Existing evidence is complex, with different experimental approaches leading to varying conclusions. Zeberg et al.^7^ reported that when expressed in *Xenopus Laevis* oocytes in vitro, none of the three variants in isolation is sufficient to alter the activation or inactivation properties of Nav1.7. The simultaneous expression of V991L and D1908G (but not of M932L and D1908G or M932L and V991L) was sufficient to cause a depolarizing shift in the voltage dependence of inactivation of the Nav1.7 channel. This was interpreted as suggestive of an interaction between V991L and D1908G. These two amino acids occupy intracellular domains in Nav1.7: V991L is in the second intracellular loop (between the second and third domain), and D1908G is in the C-terminal domain. There are several ankyrin-G binding motifs located in the second intracellular loop and these are important for the trafficking of sodium channels to the axonal initial segment and nodes of Ranvier, with potential implications in cellular localization and neuronal properties^33^. In contrast to the results of Zeberg et al.^7^, expression studies in rodent dorsal root ganglia by Faber et al.^6^ observed that the combined expression of M932L and V991L did alter channel function: although the inactivation properties were unchanged (as seen by Zeberg et al.^7^), this two amino acid combination enhanced resurgent currents (which were not characterized by Zeberg et al.^7^) and made dorsal root ganglia neurons more excitable^6^. The impact of the Neanderthal variants on channel function is probably dependent on cellular context.

In terms of association with pain traits in the population, the strong LD between the three Nav1.7 Neanderthal variants complicates distinguishing phenotypic effects they might have individually (or in various combinations). This LD reflects the fact that these variants are located on a small genomic segment (~78 Kb). As has been reported for 1000 Genomes data, in Latin Americans we observe an essentially perfect LD between M932L and V991L (separated by 4 Kb) with identical allele frequencies at these two variants and only two haplotypes present (the fully ancestral or the fully derived). In these circumstances, the effects of these two variants are impossible to distinguish in an association study. In this context, it is surprising that Zeberg et al.^7^ reported the presence in the UKBB of individuals carrying only M932L or V991L (albeit at frequencies of 8 × 10^−5^ and 4 × 10^−4^, respectively), as well as individuals carrying M932L (without V991L) and D1908G (at a frequency of 6 × 10^−5^). The rarity of these variants in Europeans and the fact that imputation in the UK Biobank data has moderate reliability for alleles with frequency <0.5% (Supplementary Fig. 4) complicates robust inferences based on them. Considering this rarity, when multiple studies on any pain trait become available, it would be useful to aggregate the evidence via meta-analyses to understand the overall impact of Neanderthal introgressed variants on the trait. Although LD makes difficult detecting independent effect for M932L-V991L and D1908G using single-locus tests, the analyses we performed based on haplotypes and number of alleles are consistent with some degree of additivity for the effects of these two loci. Thus, although strongest effect is seen for carriers of the three Neanderthal variants (consistent with Zeberg et al.^7^), our analyses also suggest that individuals carrying less than three variants also have greater pain sensitivity. In addition, the fact that in our regional introgression analyses strongest association was observed for non-coding segments raises the possibility that, other than the three amino acid changing variants altering the function of Nav1.7, Neanderthal introgression could impact regulatory elements in the region. In this respect, it is intriguing that the introgression segment with strongest association across the *SCN9A* region (segment 33, Figs. 2c and 4e) encompasses a binding site for transcription factor CEBPB (CCAAT enhancer-binding protein beta), which has been shown to regulate immunity and inflammation genes, expression of which has been shown to modulate pain-related behaviours in mice^34^.

Recent studies are providing an increasingly refined view on the evolution and phenotypic importance of Neanderthal introgression in the genome of modern humans^35^. Introgression has been associated with a range of phenotypes, including some differentiated between human populations, particularly for certain metabolic, pigmentation and immune-related traits^36–41^. Acute pain (unlike chronic pain) is adaptive in modulating behaviour and preventing further injury. Furthermore sensory neurons have a wider role in mediating host defence for instance through reflexes such as cough^42^ and the response to pathogens^43^. The length of the core introgression tract observed here (123 Kb) is within the range of proposed cases of adaptive introgression^44^. Furthermore, recent analyses point to the action of balancing selection on *SCN9A* in certain animal taxa^45^. These observations raise the intriguing possibility that Neanderthal *SCN9A* introgression could have played a role in environmental adaptation in modern humans. Evolutionary pressures on *SCN9A* are likely complex. Why Neanderthals might have had a greater pain sensitivity and whether introgression in *SCN9A* represented an advantage during human evolution remains to be determined.

## Methods

### Study sample

The CANDELA cohort consists of more than 7000 individuals that were recruited in five Latin American countries (Brazil, Chile, Colombia, Mexico and Peru) for the genetic study of non-pathological variation in the population^15^. Individuals for the QST cohort were recruited in Medellin (Colombia) via open lectures, public notice boards and distribution of flyers at Universidad de Antioquia Faculty of Odontology^21^. This cohort consists of 1963 individuals recruited in Medellín (Colombia), with a median age of 22 (range 18–45) years, of which 56.1% were female (Supplementary Data 2). Only individuals not reporting any history of medical history related to pain status were recruited for the study. This study was approved by the bioethics committee of the Odontology Faculty at the University of Antioquia (CONCEPTO-01-2013). All participants provided written informed consent.

### Phenotyping

Thresholds for six pain modalities (HPT, MPT, POST_HPT, POST_MPT, PPT and WUR; Supplementary Data 9) were obtained following our QST protocol described in detail in Schmid et al.^18^. Participants were also evaluated with the 16-Item Quick Inventory of Depressive Symptomatology^46^. Individuals reporting severe depression (score>15) were excluded from QST. All pain thresholds were measured in the forearm, except for PPT, which was assessed on the hand (thenar muscles). The distribution of the QST traits was highly skewed (Supplementary Figs. 5–8), with the occurrence of sporadic outliers. For the genetic analyses we excluded extreme outliers (over ±2 standard deviations) and performed a log transformation of the data, resulting in a more normal distribution of the traits (Supplementary Figs. 5–8). In a test–retest reliability assessment, we observed high and statistically significant intra-class correlation coefficients for all traits^18^.

### Genotyping, imputation and sample filtering

Chip genotyping of the QST cohort was achieved in two runs and on two different chips: Illumina OmniExpress (1177 individuals; 697,067 variants after genotyping quality control) and Illumina GSA (512 individuals; 683,494 variants after genotyping quality control). The GRCh37/hg19 build was used for genomic locations. Imputation was achieved independently on the two datasets, using all 2504 samples from 1000GP3 as reference. This dataset includes the 3 variants of interest (D1908G, V991L and M932L), actually at a relatively high frequency in American populations. Prior to imputation, variants monomorphic in the AMR populations of the 1000GP3 were excluded. After imputation (performed by IMPUTE2^47^), only variants with high reliability were retained. In brief, the conditions were (i) imputation quality scores ≥0.40, (ii) concordance value >0.70, (iii) gap ≤0.10 between the INFO score (an imputation quality metric returned by IMPUTE2) and concordance value, (iv) call rate ≥0.95 (after setting as uncalled genotype with maximum genotype probability <0.90) and minor allelic frequency >0.01. More details about the imputation procedure can be found in earlier studies of the CANDELA project^12^).

For quality control purposes, we merged the 1689 samples from both runs on a selection of 624,107 variants (genotyped on the OmniExpress chip and either genotyped on the GSA chip, or imputed). We ran KING v2.2.5^48^ to estimate kinships between all individuals in the merge dataset and removed 40 of them so that no pairwise kinship would be higher than 5%. Then, we performed PCA on the remaining individuals with PLINK v1.9^49^ and spotted 9 genetic outliers. We checked covariates consistency for the 1640 remaining samples; we removed 7 of them for a large discrepancy between age records at sampling and phenotyping (≥5 years) and 10 of them for inconsistency between recorded and genotyped sex, hence leaving 1623 individuals for this study.

The dataset was further enriched with CANDELA samples not phenotyped on sensitivity (710 samples from the CANDELA Colombian set intersected with QST samples). We added these samples for two purposes: (1) improving phasing quality by extending the data, and (2) assessing the distribution of *SCN9A* haplotypes over admixed Latin American populations. To that end, we applied the same individual filtering rules as above, which led to add 5971 individuals (666 Brazilians, 1765 Chileans, 1067 Colombians, 1216 Mexicans and 1257 Peruvians).

Therefore, the combined dataset included 7594 individuals (1623 Colombians with sensitivity phenotypes and 5971 non-phenotyped Latin Americans). For all these individuals, we retained 13,167 variants that were: (i) located in a 4.18 Mb window corresponding to *SCN9A* limits −/+2 Mb, (ii) retained after post-imputation filters of the GSA and the OmniExpress datasets, (iii) with MAF higher than 1%, and (iv) with variant call rate higher than 95%.

This selection included the 3 variants from Zeberg et al.^7^ (D1908G, V991L and M932L), whose INFO scores were all above 98.6% in the two datasets. We also assessed the expected concordance between imputed and true genotypes using the concordance table summarized in the IMPUTE2 run. These concordance tables are obtained by masking and imputing known genotypes. We obtained concordance rates (averaged over the two datasets) of 99.3% for D1908G and 99.2% for the two other variants. In addition, we found imputed genotypes of these 3 variants to perfectly match sequenced genotypes, for 38 samples of the QST cohort that were sequenced at high-coverage (>30×).

### Genome-wide ancestry estimations

An LD-pruned set of 93,328 autosomal SNPs was used to estimate genome-wide European, African and Native American ancestry proportions using supervised runs of ADMIXTURE^50^. Unadmixed reference parental populations included in the ADMIXTURE^50^ analyses consisted of 504 Sub-Saharan Africans (YRI, ESN, GWD, MSL, LWK) and 404 West Europeans (IBS, GBR, TSI, CEU) from 1000GP3 and 380 selected Native Americans (from North, Central, and South America). These reference individuals were selected, and their unadmixed nature was established, during a population genetics analysis conducted on the CANDELA samples with a worldwide set of diverse reference populations^25,51–55^ in Chacón-Duque et al.^16^.

### Local ancestry inference

Prior to infer local ancestry along all chromosomes, we ran analyses on the CANDELA cohort to optimize both (1) the set of reference samples of each of the 3 continental reference samples (African, European and Native American) and (2) the implementation of the inference methods. To that end, we sought to minimize two criteria: (i) the sum of differences with global ancestry estimates returned by ADMIXTURE (see above), hence assuming global ancestry is better estimated by ADMIXTURE than by local ancestry methods, and (ii) the average number of ancestry switches per individual, assuming that spurious ancestry calls will be of shorter length than true calls hence inflating the number of ancestry switches. We compared versions 1 and 2 of RFMix^24^, varying the number of expectation–maximization (EM) iterations (0, 1 or 2) and asking the software to model admixture in reference samples in EM iterations >0 (version 1 “--use-reference-panels-in-EM”, version 2 “--reanalyse-reference”). Using this option allows to properly account for admixed reference samples (such as AMR samples from 1000GP3).

We found RFMix v1 with 1 EM iteration and using 3 sets of 242 samples (total 726 samples) for each ancestral reference to produce the best results. The African reference samples were all part of the 1000GP3 (selected among the ESN, GWD, MSL and YRI populations). Most of the European and Native American samples were collected and genotyped in an earlier study of the CANDELA cohort^16^ and combined with selected samples from the 1000GP3 (IBS and TSI for the European reference; MXL and PEL for the Native American reference).

This method implementation was then applied to all 7594 study samples (QST and CANDELA cohorts) previously phased using Shapeit4^56^ (with default parameters and without reference phases). In the scope of this study, we retained the ancestry tracts located in the 4.18 Mb defining the region of interest. These ancestry tracts were used at various places in downstream analyses, noticeably to assess the per-ancestry frequency of each allele at each of the 3 variants and to determine a length threshold for introgression tracts (see below and Supplementary Note 1). In addition, RFMix outputs enhanced phases, taking advantage of local ancestry calls to detect phasing errors; we used these enhanced phases as a scaffold for phasing the imputed data over the region of interest (see below).

### Phasing of imputed data and alignment to local ancestry calls

We pre-phased the 7594 individuals (from both CANDELA and QST cohorts) of the combined dataset over the region of interest (13,167 imputed variants) using Shapeit4^56^ (with default parameters and without reference phases).

We have then aligned these pre-phases to phases obtained after running RFMix v1^24^ on the same individuals (with a set of 242 reference individuals of each continental ancestry and 1 EM iteration). Achieved with a strategy^57^ relying on Shapeit2^58^, this operation has two purposes. First, RFMix^24^ ran on chip genotypes (see above), which are ~15× less dense than imputed data, hence less prone to phase switches. Furthermore, this program enhances the phases by detecting potential phase switches based on inferred local ancestry tracts. Aligning the pre-phases to these phases is therefore expected to lower the number of phase switches. Secondly, the resulting phases are then aligned to local ancestry calls. This alignment therefore allows to estimate the frequencies of *SCN9A* haplotypes within each continental ancestry by focusing on the 12,136 chromosomes (over a total of 2 × 7594 = 15,188 chromosomes) with invariant local ancestry inferred over the studied region.

### Introgression scans

We carried out introgression scans on all phased chromosomes (*N* = 15,188) in the *SCN9A* region (gene limits ±2 Mb, 4.18 Mb in total) with the script *admixtureHMM*^59^. Using the Viterbi algorithm, this script models each chromosome as a succession of archaic or modern tracts, provided Neanderthal allele frequencies at each site in the region and for proxies of each ancestral population. We used the Vindija Neanderthal genome (Vindija 33.19^60^) as archaic proxy; this female sample is representative of the late Neanderthal population (~50 kya), who introgressed to *H. sapiens*^35^. As modern human proxy, we used the allelic frequencies of the 108 individuals from the YRI population of the 1000GP3; this Western African population does not show signs of back-migration from Eurasia, unlike Northern and Eastern African populations^61,62^. Following a protocol described earlier^13^, we retained only biallelic sites: (i) with depth ≥20 in Vindija 33.19, (ii) with filter PASS in VCFs of Vindija 33.19 and 1000GP3, (iii) with reported ancestral allele consistent throughout sources, and (iv) with identical polymorphism in both VCFs. This led to a selection of 8776 sites over the region, i.e. on average less than 500 bp between each site. Only Viterbi outputs with probability >99% were considered, leading to assign a total of 19,590 segments to archaic ancestry. A great proportion of these putative archaic tracts were rather short (10% of them below 6 Kb) and may results from spurious introgression calls. Therefore, we defined a physical length cut-off to discard short tracts, relying on the assumption that chromosomes of African origin are unlikely to carry archaic tracts. This analysis, extensively detailed in Supplementary Note 1, returned a cut-off of 23.5 Kb, a value close to that of 0.02 cM used as threshold for considering reported introgression tracts in Zeberg et al.^7^ (the recombination rate averages to 0.79 cM/Mb in this window). Applying this post-calling filter led to a final number of 12,220 archaic tracts (Supplementary Fig. 9 and Supplementary Data 10).

### Association testing

We carried out association testing with the six pain thresholds using a linear model incorporating eight covariates (sex, age, depression score and five genetic PCs) using PLINK v1.9^49^. Genotypes were: (i) the imputed genotypes at the 2 loci formed by the 3 variants (1 locus standing for D1908G variant and 1 locus standing for V991L and M932L – these two variants being called together in 99.9% of the samples of the QST cohort), (ii) these 2 loci tested jointly, (iii) the number of copies of each of the 4 haplotypes formed by these 2 loci, (iv) the sum of Neanderthal alleles carried at the 2 tested loci, or (v) the number of copies of archaic tracts at each of the 34 introgression segments (defined by an admixture mapping approach detailed here below). The single-variant case (i) was applied to the 6 pain modalities mentioned above (see section “Phenotyping”) and to mechanical pain threshold after mustard oil sensitization (POST_MPT) adjusted to pre-sensitization mechanical pain threshold MPT (denoted as Adjusted POST_MPT). This adjustment was done by adding MPT as a ninth covariate in the linear model. The four other cases (ii, iii, iv and v) were tested for association with only one pain threshold (POST_MPT).

The case (ii) is that of a joint model of association, that is, including 2 genotypic terms (one for each tested locus) in the linear model instead of a single one.

In the case (iii), the four haplotypes to test were *AB*, *Ab*, *aB* and *ab*, where *A* stands for D1908G, *B* for the paired variants V991L and M932L and lower-case letters for the corresponding ancestral alleles. In each haplotype-based test, the individual dosage of the tested haplotype was obtained by counting the copies of this haplotype carried by an individual. This way, each haplotype was compared to the union of the 3 other haplotypes, i.e. *AB* vs. {*Ab*, *aB*, *ab*}, *Ab* vs. {*AB*, *aB*, *ab*}, *aB* vs. {*AB*, *Ab*, *ab*}and *ab* vs. {*AB*, *aB*, *Ab*}. In order to keep the effect in the same direction for all tests, we have reverted the focal allele in the last test: {*AB*, *Ab*, *aB*} (i.e. any haplotype that carry at least one Neanderthal variant) was counted instead of the ancestral haplotype (*ab*). We provided details about the coding in Supplementary Data 11, along with numbers for each haplotype-based genotype. Please note that no individual homozygous for the least frequent haplotype (*aB*; 2.1% in CANDELA cohort and 0.5% in QST cohort) was observed.

In the case (iv), the linear model of association actually includes only one genotypic term, a dosage summing the Neanderthal alleles carried by an individual at the 2 loci, hence ranging from 0 (homozygous ancestral at the 2 loci) to 4 (homozygous Neanderthal at the 2 loci).

For the case (v), we tested the association of the called introgression segments with POST_MPT in an admixture mapping approach previously described in Li et al.^63^ and detailed in Supplementary Data 12. In brief, in this approach, we first code each individual genotype at each site as the number of times the individual carries an archaic tract at this position. Then, we merge consecutive sites with identical genotype distribution over all individuals retained for association testing. In order to limit similar merges of consecutive sites, we allow for nearly identical consecutive sites (less than 0.1% genotype differences) to be merged. This process therefore defines short segments of nearly unvarying distribution of ancestry across individuals (see Supplementary Data 12). For association testing, we retained only the 34 segments with archaic ancestry >1%; the retained segments are on average 17.3 Kb long.

### Adjustment for multiple testing

In this study, a total of 55 association *P* values were calculated: (i) 2 × 7 = 14 tests, for single-SNP *SCN9A* loci (Fig. 3), (ii) two tests, for the joint test of single loci (Supplementary Data 7), (iii) four tests, for 2-loci haplotypes (Fig. 4c), (iv) one test, for the sum of Neanderthal alleles over 2 loci (Fig. 5) and (v) 34 tests, for the introgression segments (Figs. 2b and 4c). All summary statistics are compiled in Supplementary Data 7.

We performed a study-wide adjustment of multiple testing, by using the false discovery rate (FDR) method with the Benjamini–Hochberg procedure^64^. The study-wide significance threshold corrected for multiple testing was calculated to be 9.75 × 10^−3^ with this method. This significance threshold was applied to all the association *P* values reported in this study.

### Reporting summary

Further information on research design is available in the Nature Portfolio Reporting Summary linked to this article.

### Supplementary information

Supplementary Information

Description of Additional Supplementary Files

Supplementary Data 1-13

Reporting Summary

## Data Availability

Raw genotype or phenotype data cannot be made available due to restrictions imposed by the ethics approval. Summary statistics for all the association tests performed in this study can be found in Supplementary Data 7. Numerical data used for the production of manuscript figures was compiled in Supplementary Data 13. All other data are available from the corresponding author on reasonable request. The high-coverage Neanderthal sequence of Vindija33.19 was downloaded from http://cdna.eva.mpg.de/neandertal/Vindija/VCF/Vindija33.19/chr2_mq25_mapab100.vcf.gz. Allelic frequencies in the Yoruba (YRI) population were obtained from the 1000 Genomes Project Phase III, downloaded from http://ftp.1000genomes.ebi.ac.uk/vol1/ftp/release/20130502/ALL.chr2.phase3_shapeit2_mvncall_integrated_v5b.20130502.genotypes.vcf.gz.
