# Supplementary material for: Neanderthal introgression in SCN9A impacts mechanical pain sensitivity
Source: Commun Biol. 2023 Oct 10;6:958. doi: 10.1038/s42003-023-05286-z (PMC10564861; doi:10.1038/s42003-023-05286-z)
Supplement: Supplementary file 1 — Supplementary Information [file 42003_2023_5286_MOESM1_ESM.pdf]

Supplementary Information:

Neanderthal introgression in *SCN9A* impacts mechanical pain sensitivity

Table of contents

Supplementary Note 1. Post-calling filtering of introgression tracts 2
- Selection of chromosomes of invariant local ancestry 2
- Distribution of lengths of introgressed tracts per ancestry 2

Supplementary Figure 1. Admixture plots 3

Supplementary Figure 2. Frequencies of the main haplotypes in the 1000 Genomes Project Phase III and in the CANDELA cohort 4

Supplementary Figure 3. Neanderthal introgression profile around *SCN9A* per cohort 5

Supplementary Figure 4. Imputation reliability in the UK Biobank, stratified by minor allele frequency 6

Supplementary Figure 5. Distributions of the heat pain threshold (HPT) and post-sensitization HPT (POST_HPT) 7

Supplementary Figure 6. Distributions of the mechanical pain threshold (MPT) and post-sensitization MPT (POST_MPT) 8

Supplementary Figure 7. Distribution of the pressure pain threshold (PPT) 9

Supplementary Figure 8. Distribution of the wind-up ratio (WUR) 10

Supplementary Figure 9. Distribution of the lengths of introgression tracts detected in each continental ancestry 11

Supplementary references 12

Supplementary Note 1. Post-calling filtering of introgression tracts

Short introgression tracts, although called at high confidence (>99%), are likely to result from punctual differentiation of alleles and therefore be spurious inferences of archaic introgression. In order to filter out these tracts, we targeted those detected on chromosomes segments that were inferred to be of African descent (hence, in which Neanderthal admixture is unlikely) and set a threshold at the 95th percentile of their distribution of lengths. We provide more details on this filtering here below.

Selection of chromosomes of invariant local ancestry

For each chromosome of each of the study individual (N = 7,594 * 2 = 15,188), we first determined if the study region (SCN9A +/- 2Mb, i.e. chr2:165,051,695-169,232,511 – hg19 coordinates) was inferred to be in continuous segment of the same ancestry when analysed by RFMix v1[1]. This analysis had previously been performed on all CANDELA individuals (unpublished results) and further applied to individuals phenotyped in the present study. RFMix was applied using 3 reference populations of 242 samples (one population per continental ancestry: African, European and Native American), with default parameters and on 1,373 chip SNPs in that region.
We found 512, 6,523 and 5,101 chromosomes (total 11,736; ~77% of the study population) to respectively have only African, European and Native American ancestry over the SCN9A region. The proportions given by these numbers (respectively 4, 54 and 42%) are close to the average ancestral proportions in the CANDELA cohort (respectively 4, 50 and 46%)

Distribution of lengths of introgressed tracts per ancestry

We have then collected all introgression tracts detected on these 11,736 chromosomes and pooled the tracts per ancestry. In Supplementary Figure 9 and in the top half of Supplementary Data 10, we give, the number of introgression tracts called and the distribution of lengths of tracts detected in each continental ancestry. Over the 512 African chromosomes scanned, we detected >100 introgression tracts on average 10 times shorter than those detected in Native American chromosomes. Most of these tracts are however likely to be false calls, as African individuals are very unlikely to have introgressed segment from Neanderthal. We therefore decided to arbitrarily consider that any tract shorter than 23.5 Kb (corresponding to the 95th percentile of the length distribution in African chromosomes) should be discarded in any ancestry.

Doing so, we mostly impacted calls in African and European chromosomes, reducing their number by the same rate (-97% - second half of Supplementary Data 10) while not shifting much the average tract length in Native American chromosomes (from 102 to 124 Kb).

Supplementary Figure 1. Admixture plots

The plots below show the proportions of the 3 continental ancestries (EUR: European, AFR: African and NAT: Native American – estimated using RFMix v1[1]) in stacked bins ordered by ascending proportion of European ancestry. Figure **(a)** distinguishes the 1,623 samples with QST phenotypes (including 710 samples from the CANDELA project) that were retained for association studies (left) from the 5,971 samples selected from the CANDELA project. The ancestry proportions of these samples vary between country: in Figure **(b)**, we show these 5,971 samples separated into countries.

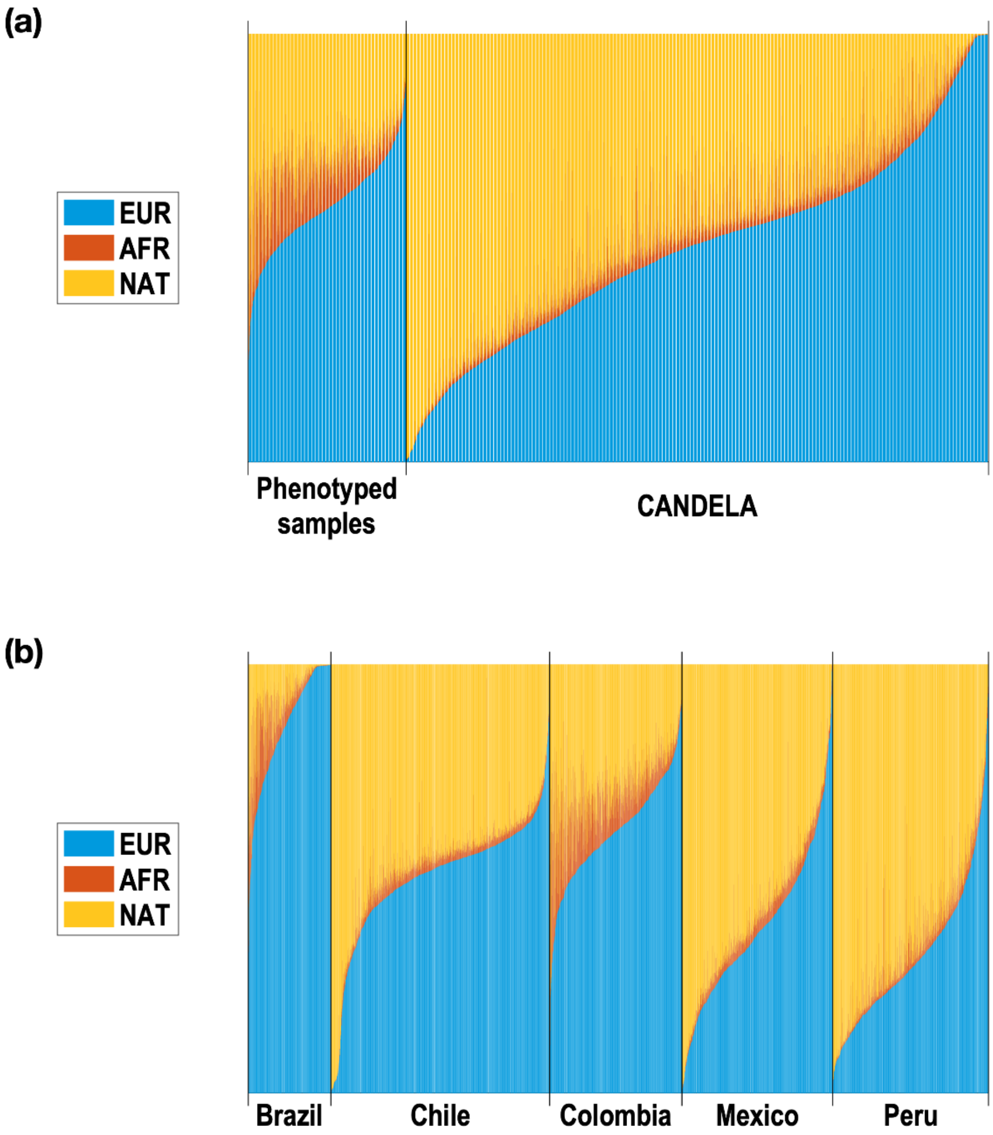

Supplementary Figure 2. Frequencies of the main haplotypes in the 1000 Genomes Project Phase III and in the CANDELA cohort

In this figure, we plot the 1000GP3 vs. CANDELA frequencies for the 4 main haplotypes formed by the 3 variants of interest for the populations of 3 countries of the CANDELA cohort that have proxy populations in the 1000GP3 (Colombia, Mexico and Peru). The 4 haplotypes are :

- Ancestral (T-C-T, i.e. no mutation)
- Derived (C-A-G, i.e. D1908G + V991L + M932L)
- Partially derived (C-C-T, i.e. D1908G alone)
- Partially derived (T-A-G, i.e. V991L + M932L)

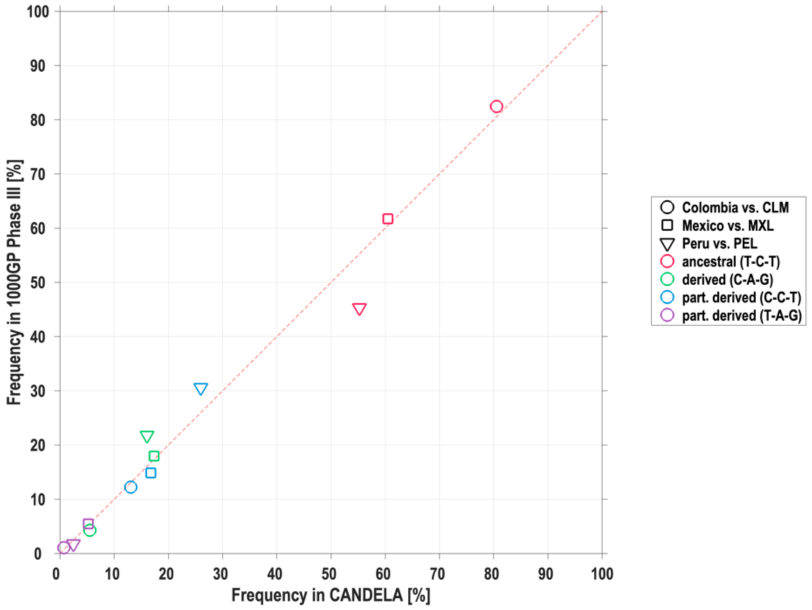

Supplementary Figure 3. Neanderthal introgression profile around *SCN9A* per cohort

The three subplots show (in violet) the frequency of Neanderthal introgression tracts (piling-up tracts across individuals) over a 700 Kb window around the *SCN9A* gene, in the QST (N = 1,623), CANDELA (N = 5,971) and merged (N = 7,594) cohorts. This window covers the physical coordinates 2:166,000,000-167,200,000 of the GRCh37 (hg19) reference genome. The positions of the 3 variants of interest are indicated by dashed blue lines. Introgression profile of the QST cohort is also reproduced in Figure 2a of the manuscript.

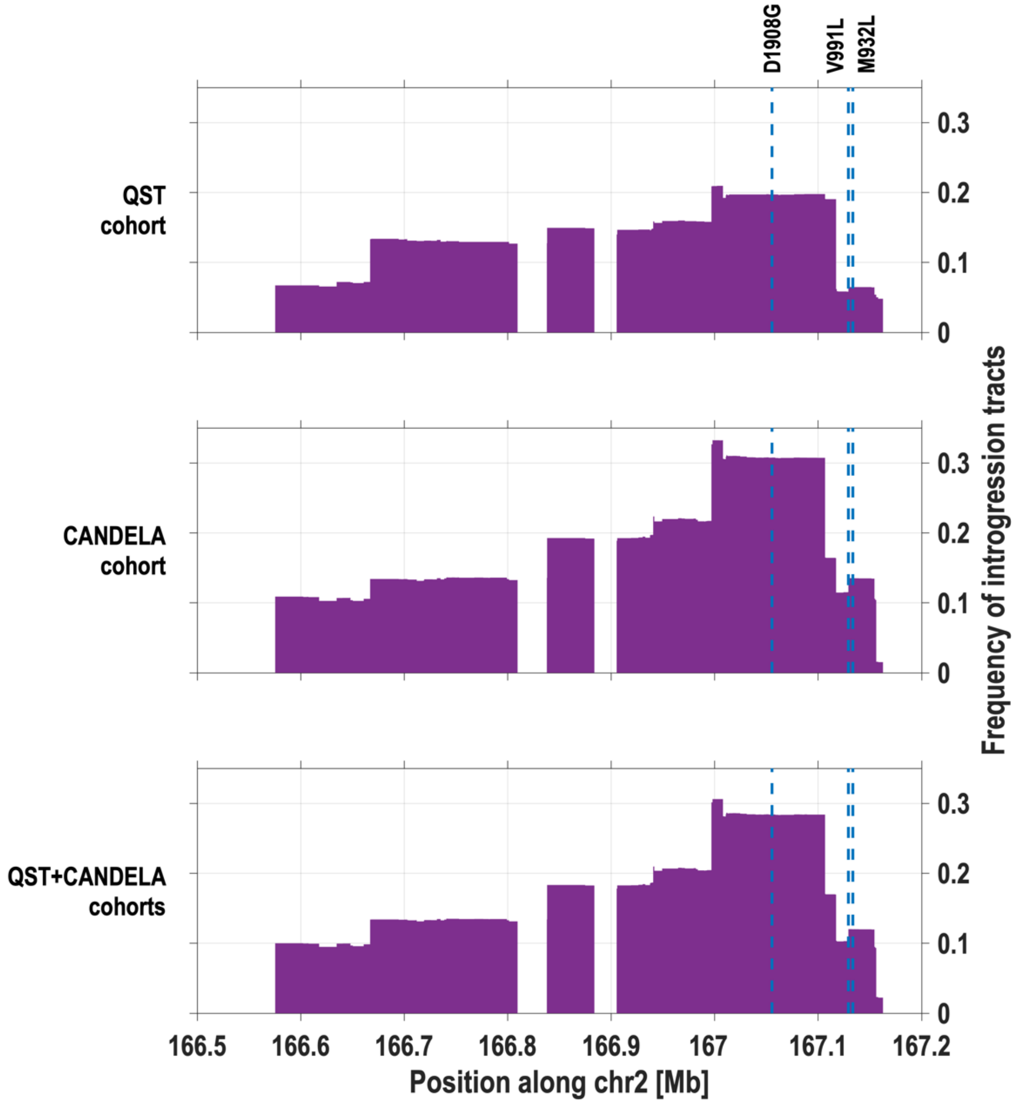

Supplementary Figure 4. Imputation reliability in the UK Biobank, stratified by minor allele frequency

The below figure was copied from the "UK Biobank Phasing and Imputation Documentation"[2] (a document publicly available at https://biobank.ndph.ox.ac.uk/showcase/showcase/docs/impute_ukb_v1.pdf - downloaded on 2023-05-11) It aims at comparing the imputation performance of various genotyping arrays, including the Affymetrix UKB array ("Affy UK Biobank", in purple). Although this array outperforms other arrays of similar density, it shows moderate reliabilities in the range of allelic frequency corresponding to the 3 Neanderthal variants of interest (0.2-0.5%).

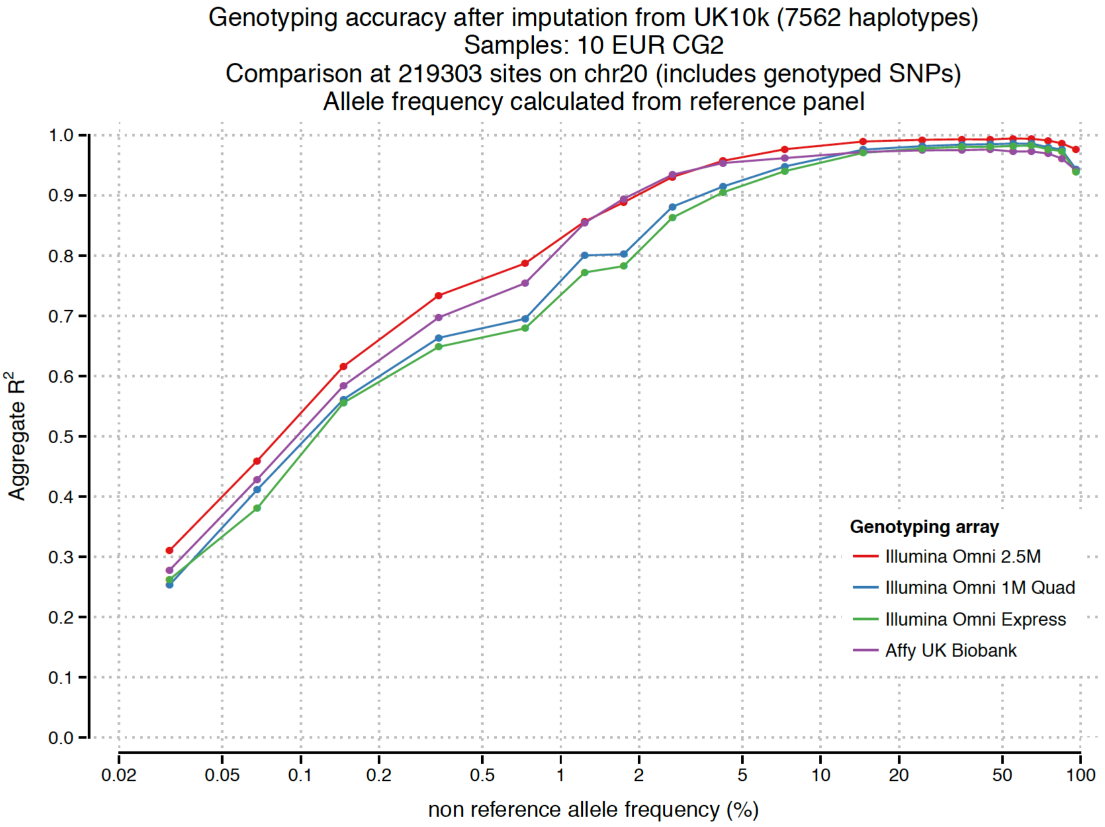

Supplementary Figure 5. Distributions of the heat pain threshold (HPT) and post-sensitization HPT (POST_HPT)

The plots below show the distributions of heat pain threshold (HPT) and post-sensitisation HPT (POST_HPT) and the colored dots on the X axis represent the sigma intervals. We log-transformed the corrected pain traits and observed a more normal distribution.

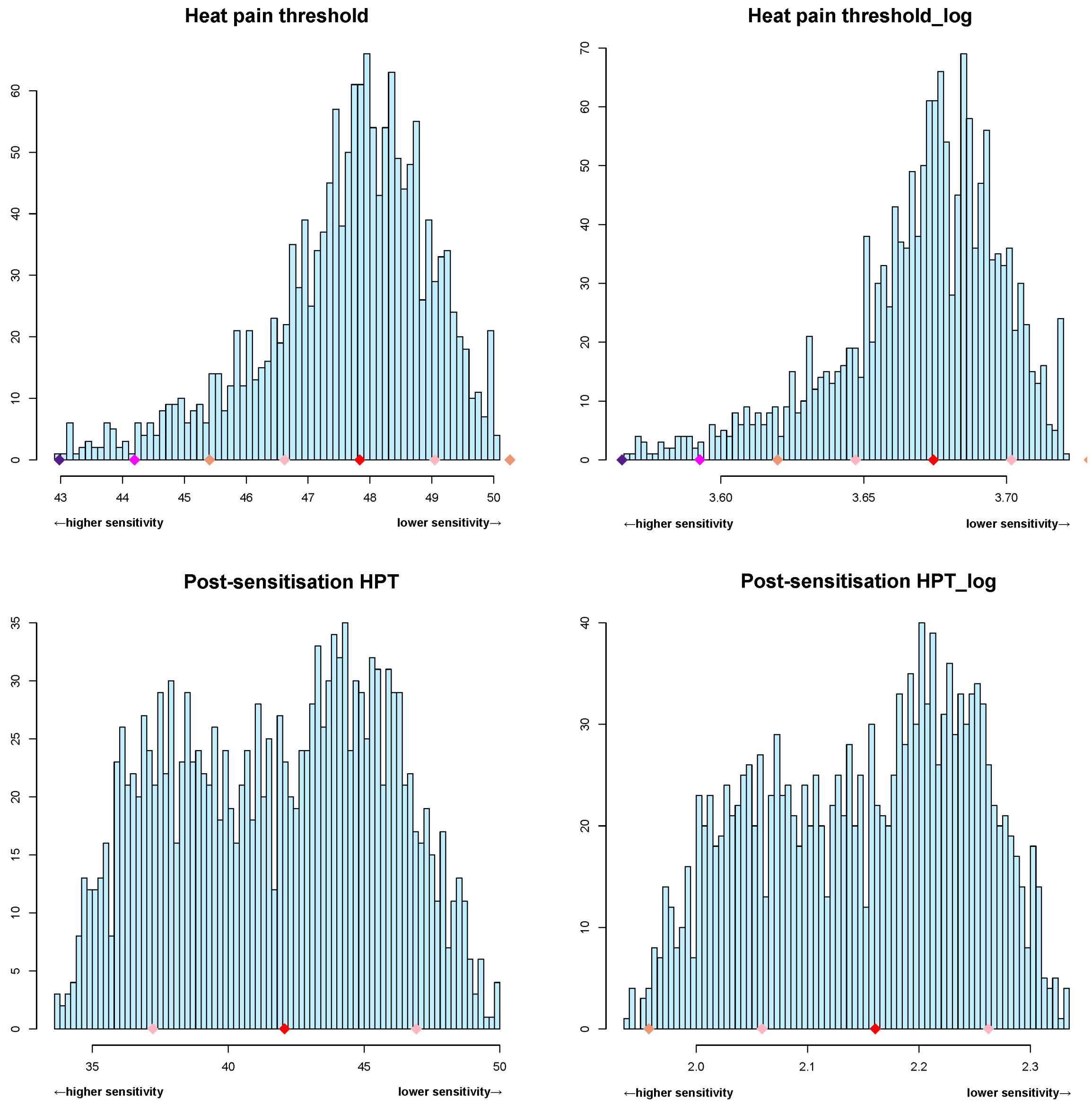

Supplementary Figure 6. Distributions of the mechanical pain threshold (MPT) and post-sensitization MPT (POST_MPT)

The plots below show the distributions of mechanical pain threshold (MPT) and post-sensitisation MPT (POST_MPT) the colored dots on the X axis represent the sigma intervals. We log-transformed the corrected pain traits and observed a more normal distribution.

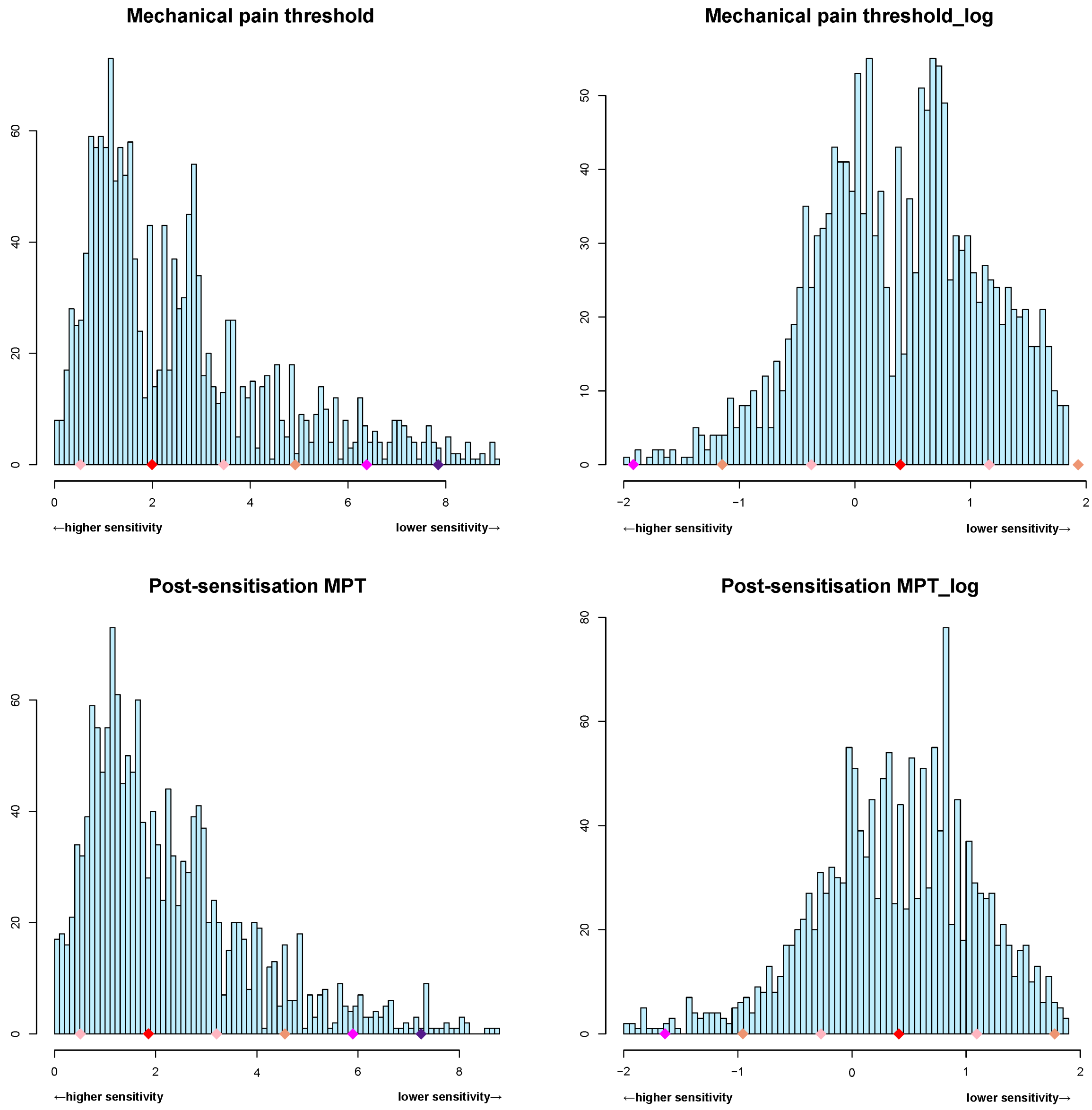

Supplementary Figure 7. Distribution of the pressure pain threshold (PPT)

The plots below show the distributions of pressure pain threshold (PPT) and the colored dots on the X axis represent the sigma intervals. We log-transformed the corrected pain trait and observed a more normal distribution.

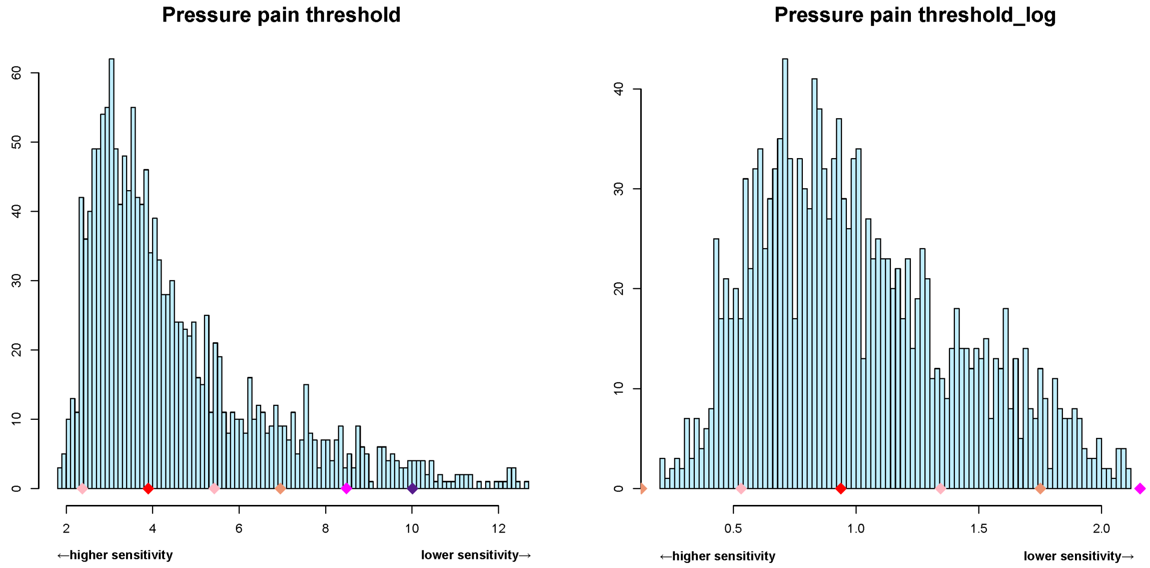

Supplementary Figure 8. Distribution of the wind-up ratio (WUR)

The plots below show the distributions of wind-up ratio (WUR) and the colored dots on the X axis represent the sigma intervals. We log-transformed the corrected pain trait and observed a more normal distribution.

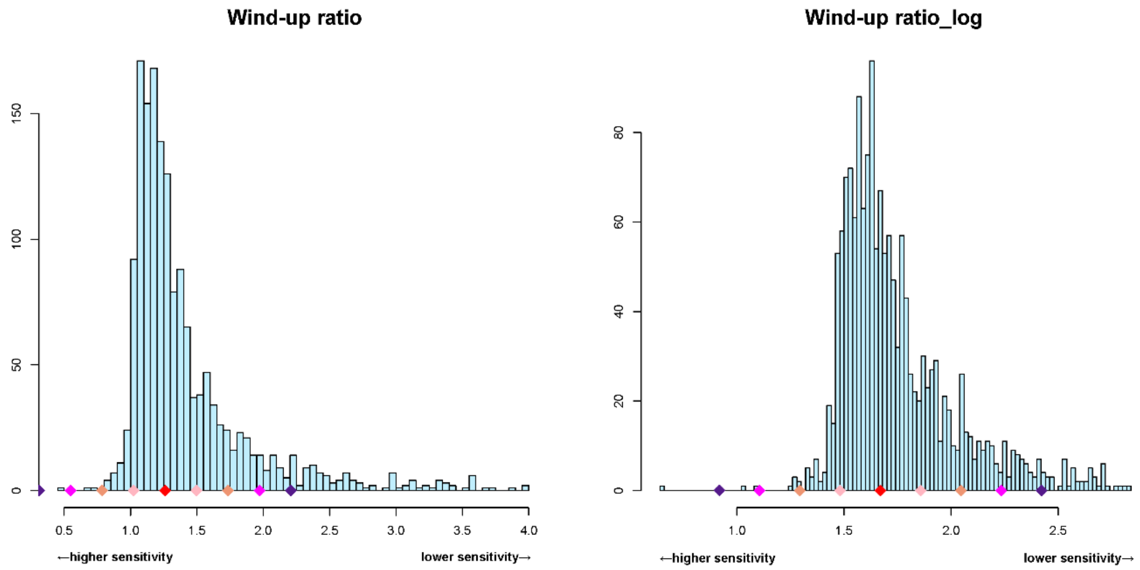

Supplementary Figure 9. Distribution of the lengths of introgression tracts detected in each continental ancestry

The dashed green line indicates the threshold used for considering calls as spurious (23.5 Kb).

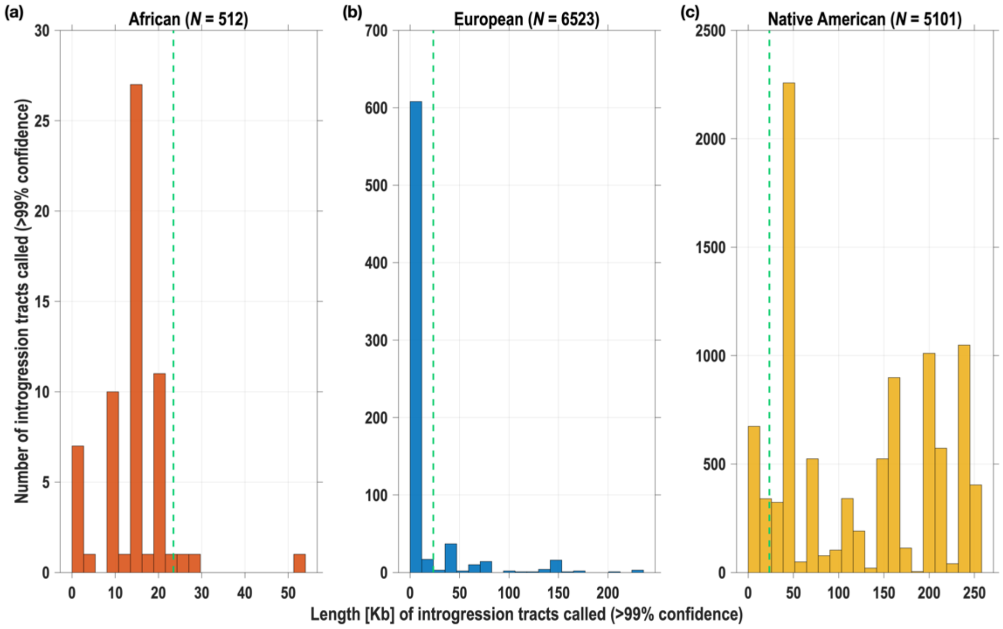

Supplementary references

1. Maples, B. K., Gravel, S., Kenny, E. E. & Bustamante, C. D. RFMix: A Discriminative Modeling Approach for Rapid and Robust Local-Ancestry Inference. *The American Journal of Human Genetics* **93**, 278–288 (2013).
2. Marchini, J. UK Biobank Phasing and Imputation Documentation, Version 1.2., Department of Statistics, University of Oxford on behalf of UK Biobank (2015). (PDF downloaded on 2023-05-11)
