# Supplementary material for: Neanderthal introgression in SCN9A impacts mechanical pain sensitivity
Source: Commun Biol. 2023 Oct 10;6:958. doi: 10.1038/s42003-023-05286-z (PMC10564861; doi:10.1038/s42003-023-05286-z)
Supplement: Supplementary file 2 — Description of Additional Supplementary Files [file 42003_2023_5286_MOESM2_ESM.pdf]

Description of Additional Supplementary Files

File Name: Supplementary Data 1
Description: Mean continental ancestry estimates per cohort and country

File Name: Supplementary Data 2
Description: Covariates of QST cohort

File Name: Supplementary Data 3
Description: Correlation of experimental pain traits with covariates

File Name: Supplementary Data 4
Description: Allelic frequencies of the 3 Neanderthal variants in various cohorts and populations

File Name: Supplementary Data 5
Description: Assessment of linkage disequilibrium between the 3 variants

File Name: Supplementary Data 6
Description: Frequencies of the 4 most common 3-SNP haplotypes, per cohort and country

File Name: Supplementary Data 7
Description: Summary statistics of all association tests performed in the study

File Name: Supplementary Data 8
Description: Physical coordinates, frequencies and association P-values of tested introgression segments

File Name: Supplementary Data 9
Description: Description of 6 QST phenotypes

File Name: Supplementary Data 10
Description: Distribution of length of introgression tracts detected in each continental ancestry

File Name: Supplementary Data 11
Description: Codings and counts of genotype for the haplotype-based analyses

File Name: Supplementary Data 12
Description: Admixture mapping approach: definition of introgression segments from called tracts

File Name: Supplementary Data 13
Description: Source data for manuscript figures
