# Supplementary material for: Neanderthal introgression in SCN9A impacts mechanical pain sensitivity
Source: Commun Biol. 2023 Oct 10;6:958. doi: 10.1038/s42003-023-05286-z (PMC10564861; doi:10.1038/s42003-023-05286-z)
Supplement: Supplementary file 4 — Reporting Summary [file 42003_2023_5286_MOESM4_ESM.pdf]

nature portfolio

Corresponding author(s): Andrés Ruiz-Linares

Last updated by author(s): Aug 23, 2023

Reporting Summary

Nature Portfolio wishes to improve the reproducibility of the work that we publish. This form provides structure for consistency and transparency in reporting. For further information on Nature Portfolio policies, see our Editorial Policies and the Editorial Policy Checklist.

Statistics

For all statistical analyses, confirm that the following items are present in the figure legend, table legend, main text, or Methods section.

n/a	Confirmed	
☐	☒	The exact sample size (*n*) for each experimental group/condition, given as a discrete number and unit of measurement
☐	☒	A statement on whether measurements were taken from distinct samples or whether the same sample was measured repeatedly
☐	☒	The statistical test(s) used AND whether they are one- or two-sided<br>*Only common tests should be described solely by name; describe more complex techniques in the Methods section.*
☐	☒	A description of all covariates tested
☐	☒	A description of any assumptions or corrections, such as tests of normality and adjustment for multiple comparisons
☐	☒	A full description of the statistical parameters including central tendency (e.g. means) or other basic estimates (e.g. regression coefficient) AND variation (e.g. standard deviation) or associated estimates of uncertainty (e.g. confidence intervals)
☐	☒	For null hypothesis testing, the test statistic (e.g. *F*, *t*, *r*) with confidence intervals, effect sizes, degrees of freedom and *P* value noted<br>*Give P values as exact values whenever suitable.*
☒	☐	For Bayesian analysis, information on the choice of priors and Markov chain Monte Carlo settings
☒	☐	For hierarchical and complex designs, identification of the appropriate level for tests and full reporting of outcomes
☐	☒	Estimates of effect sizes (e.g. Cohen's *d*, Pearson's *r*), indicating how they were calculated

Our web collection on statistics for biologists contains articles on many of the points above.

Software and code

Policy information about availability of computer code

Data collection	N.A.
Data analysis	VCFtools v0.1.13 was used to merge data sets and filter variants. Shapeit4, Shapeit2, RFMix v1 and admixtureHMM were used for chromosome reconstruction analyses. Plink v1.9 and Matlab R2019a were used for association analyses.

For manuscripts utilizing custom algorithms or software that are central to the research but not yet described in published literature, software must be made available to editors and reviewers. We strongly encourage code deposition in a community repository (e.g. GitHub). See the Nature Portfolio guidelines for submitting code & software for further information.

Data

Policy information about availability of data

All manuscripts must include a data availability statement. This statement should provide the following information, where applicable:

- Accession codes, unique identifiers, or web links for publicly available datasets
- A description of any restrictions on data availability
- For clinical datasets or third party data, please ensure that the statement adheres to our policy

Raw genotype or phenotype data cannot be made available due to restrictions imposed by the ethics approval. Summary statistics for all the association tests performed in this study can be found in Supplementary Data 7. Numerical data used for the production of manuscript figures was compiled in Supplementary Data 12. All other data are available from the corresponding author on reasonable request.

The high-coverage Neanderthal sequence of Vindija33.19 was downloaded from http://cdna.eva.mpg.de/neandertal/Vindija/VCF/Vindija33.19/chr2_mq25_mapab100.vcf.gz. Allelic frequencies in the Yoruba (YRI) population were obtained from the 1000 Genomes Project Phase III, downloaded from http://ftp.1000genomes.ebi.ac.uk/vol1/ftp/release/20130502/ALL.chr2.phase3_shapeit2_mvncall_integrated_v5b.20130502.genotypes.vcf.gz.

Research involving human participants, their data, or biological material

Policy information about studies with human participants or human data. See also policy information about sex, gender (identity/presentation), and sexual orientation and race, ethnicity and racism.

Reporting on sex and gender	Sex (as biological attribute, and obtained from chromosome X and Y genotyping) was correlated to various tested phenotypes in this study and therefore used in the model that tests association between these phenotypes and genetic variants of relevance for the study.<br>The findings are not limited to any sex or gender.
Reporting on race, ethnicity, or other socially relevant groupings	N.A.
Population characteristics	Two cohorts of adult subjects of both sexes aged between 18 and 45 years old were recruited (the CANDELA and QST cohorts)
Recruitment	For the CANDELA cohort, Recruitment took place mainly in five locations: México City (México), Medellín (Colombia), Lima (Perú), Arica (Chile) and Porto Alegre (Brazil). With the exception of Chile, most subjects recruited in these cities were students and staff from the universities participating in this research. In Chile about 2/3 of the subjects recruited were professional soldiers. In Brazil ~10% of samples were collected in smaller towns of the states of Rio Grande do Sul, Bahia and Rondonia.<br>For the QST cohort (partially overlapping CANDELA cohort), recruitment took place in Medellin (Colombia).
Ethics oversight	For the CANDELA cohort, ethics approvals were obtained from Universidad Nacional Autónoma de México (México), Universidad de Antioquia (Colombia), Universidad Perúana Cayetano Heredia (Perú), Universidad de Tarapacá (Chile), Universidade Federal do Rio Grande do Sul (Brazil), and University College London (UK).<br>For the QST cohort, the study was also approved by the bioethics committee of the Odontology Faculty at the University of Antioquia (CONCEPTO-01-2013).<br>All participant provided written informed consent.

Note that full information on the approval of the study protocol must also be provided in the manuscript.

Field-specific reporting

Please select the one below that is the best fit for your research. If you are not sure, read the appropriate sections before making your selection.

☒ Life sciences ☐ Behavioural & social sciences ☐ Ecological, evolutionary & environmental sciences

For a reference copy of the document with all sections, see nature.com/documents/nr-reporting-summary-flat.pdf

Life sciences study design

All studies must disclose on these points even when the disclosure is negative.

Sample size	Total sample size is 7,594: for the QST cohort, 1,623 Colombians and for the CANDELA cohort, a total of 5,971 participants from 5 countries (Colombia, Brazil, Chile, Mexico and Peru)
Data exclusions	Samples failing quality controls and/or genetic outliers to the population range were excluded from association analyses.
Replication	This study replicates a previous association between 3 genetic variants in SCN9A gene and sensitivity to pain (doi.org/10.1016/j.cub.2020.06.045). Replication in a third-party dataset was not assessed.
Randomization	Not applicable: participants were recruited in a convenience sampling, hence not selected based on tested phenotypes.
Blinding	Investigators were blinded to group allocation during data collection and analysis

Reporting for specific materials, systems and methods

We require information from authors about some types of materials, experimental systems and methods used in many studies. Here, indicate whether each material, system or method listed is relevant to your study. If you are not sure if a list item applies to your research, read the appropriate section before selecting a response.

Materials & experimental systems

n/a	Involved in the study
☒	☐ Antibodies
☒	☐ Eukaryotic cell lines
☒	☐ Palaeontology and archaeology
☒	☐ Animals and other organisms
☒	☐ Clinical data
☒	☐ Dual use research of concern
☒	☐ Plants

Methods

n/a	Involved in the study
☒	☐ ChIP-seq
☒	☐ Flow cytometry
☒	☐ MRI-based neuroimaging
